# New and noteworthy boletes from subtropical and tropical China

**DOI:** 10.3897/mycokeys.46.31470

**Published:** 2019-02-18

**Authors:** Hui Chai, Zhi-Qun Liang, Rou Xue, Shuai Jiang, Shi-Hong Luo, Yong Wang, Lu-Ling Wu, Li-Ping Tang, Yun Chen, Deng Hong, Nian-Kai Zeng

**Affiliations:** 1 College of Pharmacy-Transgenic Laboratory, Hainan Medical University, Haikou 571199, China Hainan Medical University Haikou China; 2 College of Materials and Chemistry Engineering, Hainan University, Haikou 570228, China Hainan University Haikou China; 3 Hainan Yinggeling National Nature Reserve, Baisha, 572800, China Hainan Yinggeling National Nature Reserve Baisha China; 4 College of Bioscience and Biotechnology, Shenyang Agricultural University, Shenyang 110866, China Shenyang Agricultural University Shenyang China; 5 School of Pharmaceutical Sciences and Yunnan Key Laboratory of Pharmacology for Natural Products, Kunming Medical University, Kunming, 650500, China Kunming Medical University Kunming China

**Keywords:** Molecular phylogeny, morphology, new taxa, taxonomy

## Abstract

The morphology, ecology, and phylogenetic relationships of specimens of the family Boletaceae from subtropical and tropical China were investigated. Four species, *Butyriboletushuangnianlaii*, *Lanmaoamacrocarpa*, *Neoboletusmultipunctatus*, and *Sutoriussubrufus*, are new to science. *Chalciporusradiatus* and *Caloboletusxiangtoushanensis* are redescribed. *Caloboletusguanyui* is proposed to replace *Boletusquercinus* Hongo, an illegitimate later homonym. The recently described *Tylopiluscallainus* is synonymized with the Japanese *Boletusvirescens*, and the new combination *T.virescens* (Har. Takah. & Taneyama) N.K. Zeng et al. is proposed. Moreover, *Neoboletus* is treated as an independent genus based on evidence from morphology and molecular phylogenetic data in the present study, and many previously described taxa of *Sutorius* are recombined into *Neoboletus*: *N.ferrugineus* (G. Wu et al.) N.K. Zeng et al., *N.flavidus* (G. Wu & Zhu L. Yang) N.K. Zeng et al., *N.hainanensis* (T.H. Li & M. Zang) N.K. Zeng et al., *N.obscureumbrinus* (Hongo) N.K. Zeng et al., *N.rubriporus* (G. Wu & Zhu L. Yang) N.K. Zeng et al., *N.sanguineoides* (G. Wu & Zhu L. Yang) N.K. Zeng et al. , *N.sanguineus* (G. Wu & Zhu L. Yang) N.K. Zeng et al., and *N.tomentulosus* (M. Zang et al.) N.K. Zeng et al.

## Introduction

Boletaceae Chevall. (Boletales) is a large, cosmopolitan family with abundant species. Many of them are interesting and important for their mycorrhizal relationships with trees, edibility, medicinal value, and toxicity (Wang et al. 2004; Roman et al. 2005; Wu et al. 2013; Chen et al. 2016). In China, species of Boletaceae have received much attention by mycologists, and many taxa have been discovered across the country (Chiu 1948; Zang 2013; Zeng et al. 2013, 2016, 2017; Liang et al. 2016, 2017; Wu et al. 2016a). However, the diversity of species still remains poorly known in subtropical and tropical China, a biodiversity hotspot. During field trips in the past several years, many collections of boletes have been made in subtropical and tropical China. Evidence from morphology, molecular phylogenetic analyses, and ecological data indicate that these collections belong to *Butyriboletus* D. Arora & J.L.Frank, *Caloboletus* Vizzini, *Chalciporus* Bataille, *Lanmaoa* G. Wu & Zhu L. Yang, *Neoboletus* Gelardi et al., *Sutorius* Halling et al., and *Tylopilus* P. Karst. Thus, they are described/redescribed in an effort to (i) further demonstrate the species diversity in subtropical and tropical China, (ii) resolve some taxonomic quandaries in Boletaceae

## Materials and methods

### Abbreviations of generic names used in the study

The abbreviations of *Boletus*, *Butyriboletus*, *Caloboletus*, *Chalciporus*, *Crocinoboletus*, *Lanmaoa*, *Neoboletus*, *Sutorius*, *Tylopilus* mentioned in this work are *B.*, *But.*, *C.*, *Ch.*, *Cr.*, *L.*, *N.*, *S.* and *T.*, respectively.

### Collection sites and sampling

Specimens were collected from subtropical and tropical China including Hainan and Fujian Provinces. Specimens examined are deposited in the Fungal Herbarium of Hainan Medical University (FHMU), Haikou City, Hainan Province, China, the Herbarium of Cryptogams, Kunming Institute of Botany, Chinese Academy of Sciences (HKAS), and the Mycological Herbarium of Pharmacy College, Kunming Medical University (MHKMU).

### Morphological studies

The macroscopic descriptions are based on detailed notes and photographs taken from fresh basidiomata. Color codes are from Kornerup and Wanscher (1981). Sections of the pileipellis were cut radial-perpendicularly and halfway between the center and margin of the pileus. Sections of the stipitipellis were taken from the middle part along the longitudinal axis of the stipe. Five percent KOH was used as a mounting medium for microscopic studies. All microscopic structures were drawn by freehand from rehydrated material. The number of measured basidiospores is given as *n*/*m*/*p*, where *n* represent the total number of basidiospores measured from *m* basidiomata of *p* collections. Dimensions of basidiospores are given as (*a*)*b* – *c* (*d*), where the range *b* – *c* represents a minimum of 90% of the measured values (5^th^ to 95^th^ percentile), and extreme values (*a* and *d*), whenever present (*a* < 5^th^ percentile, *d >* 95^th^ percentile), are in parentheses. *Q* refers to the length/width ratio of basidiospores; *Q*_m_ refers to the average *Q* of basidiospores and is given with a sample standard deviation.

### DNA extraction, primers, PCR and sequencing

Total genomic DNA was obtained with Plant Genomic DNA Kit (TIANGEN Company, China) from materials dried with silica gel according to the manufacturer’s instructions. The primers used for amplifying the nuclear ribosomal large subunit RNA (28S) were LROR/LR5 (Vilgalys and Hester 1990; James et al. 2006), ITS5/ITS4 (White et al. 1990) for the nuclear rDNA region encompassing the internal transcribed spacers 1 and 2, along with the 5.8S rDNA (ITS), the translation elongation factor 1-α gene (*tef1*) with 983F/1567R (Rehner and Buckley 2005) and the RNA polymerase II second largest subunit gene (*rpb*2) with RPB2-B-F1/RPB2-B-R (Wu et al. 2014). PCR products were checked in 1% (w/v) agarose gels, and positive reactions with a bright single band were purified and directly sequenced using an ABI 3730xl DNA Analyzer (Guangzhou Branch of BGI, China) with the same primers used for PCR amplifications. Assembled sequences were deposited in GenBank (Table 1).

**Table 1.** Taxa, vouchers, locations, and GenBank accession numbers of DNA sequences used in this study.

Taxon	Voucher	Locality	28S	ITS	*tef1*	*rpb*2	References
* Baorangia pseudocalopus *	HKAS63607	Yunnan, SW China	KF112355	–	KF112167	–	Wu et al. 2014
* Baorangia pseudocalopus *	HKAS75081	Yunnan, SW China	KF112356	–	KF112168	–	Wu et al. 2014
* Butyriboletus abieticola *	Arora11087	California, USA	KC184413	KC184412	–	–	Arora and Frank 2014
* Butyriboletus appendiculatus *	Bap1	Germany	AF456837	KJ419923	JQ327025	–	Binder and Bresinsky 2002
* Butyriboletus appendiculatus *	BR50200893390-25	Meise, Belgium	KT002609	KT002598	KT002633	–	Zhao et al. 2015
* Butyriboletus appendiculatus *	BR50200892955-50	Zoniënwoud, Belgium	KJ605677	KJ605668	KJ619472	KP055030	Zhao et al. 2014a
* Butyriboletus appendiculatus *	MB000286	Germany	KT002610	KT002599	KT002634	–	Zhao et al. 2015
* Butyriboletus autumniregius *	Arora11108	California, USA	KC184424	KC184423	–	–	Arora and Frank 2014
* Butyriboletus brunneus *	NY00013631	Connecticut, USA	KT002611	KT002600	KT002635	–	Zhao et al. 2015
* Butyriboletus fechtneri *	AT2003097	–	KF030270	KC584784	–	–	Nuhn et al. 2013
* Butyriboletus frostii *	JLF2548	New Hampshire, USA	–	KC812303	–	–	Arora and Frank 2014
* Butyriboletus frostii *	NY815462	Costa Rica	JQ924342	–	KF112164	KF112675	Wu et al. 2014
* Butyriboletus hainanensis *	N.K. Zeng 1197 (FHMU 2410)	Hainan, southern China	KU961651	KU961653	–	KU961658	Liang et al. 2016
* Butyriboletus hainanensis *	N.K. Zeng 2418 (FHMU 2437)	Hainan, southern China	KU961652	KU961654	KU961656	KX453856	Liang et al. 2016
* Butyriboletus huangnianlaii *	N.K. Zeng 3245 (FHMU 2206)	Fujian, SE China	**MH879688**	**MH885350**	**MH879717**	**MH879740**	this study
* Butyriboletus huangnianlaii *	N.K. Zeng 3246 (FHMU 2207)	Fujian, SE China	**MH879689**	**MH885351**	**MH879718**	**MH879741**	this study
* Butyriboletus peckii *	3959	Tennessee, USA	JQ326999	–	JQ327026	–	Halling et al. 2012
* Butyriboletus persolidus *	Arora11110	California, USA	–	KC184444	–	–	Arora and Frank 2014
* Butyriboletus primiregius *	DBB00606	Dunsmuir, California, USA	KC184451	–	–	–	Arora and Frank 2014
* Butyriboletus pseudoregius *	BR50201618465-02	Eprave, Belgium	KT002613	KT002602	KT002637	–	Zhao et al. 2015
* Butyriboletus pseudoregius *	BR50201533559-51	Meise, Belgium	KT002614	KT002603	KT002638	–	Zhao et al. 2015
* Butyriboletus pseudospeciosus *	HKAS59467	Yunnan, SW China	KF112331	–	KF112176	KF112672	Wu et al. 2014
* Butyriboletus pseudospeciosus *	HKAS63513	Yunnan, SW China	KT990541	–	KT990743	KT990380	Wu et al. 2016a
* Butyriboletus pseudospeciosus *	HKAS63596	Yunnan, SW China	KT990542	–	KT990744	KT990381	Wu et al. 2016a
* Butyriboletus pseudospeciosus *	N.K. Zeng 2127 (FHMU 1391)	Yunnan, SW China	**MH879687**	**MH885349**	**MH879716**	–	this study
* Butyriboletus pseudoregius *	MG383a	Lazio, Italy	–	KC184458	–	–	Arora and Frank 2014
* Butyriboletus pulchriceps *	DS4514	Arizona, USA	KF030261	–	KF030409	–	Nuhn et al. 2013
* Butyriboletus pulchriceps *	R. Chapman 0945	Arizona, USA	KT002615	KT002604	KT002639	–	Zhao et al. 2015
* Butyriboletus querciregius *	Arora11100	California, USA	–	KC184461	–	–	Arora and Frank 2014
* Butyriboletus regius *	MB000287	Germany	KT002616	KT002605	KT002640	–	Zhao et al. 2015
* Butyriboletus regius *	MG408a	Lazio, Italy	KC584790	KC584789	–	–	Arora and Frank 2014
* Butyriboletus regius *	PRM:923465	Czech Rep.	KJ419931	KJ419920	–	–	Šutara et al. 2014
* Butyriboletus roseoflavus *	Arora11054	Yunnan, SW China	KC184435	KC184434	–	–	Arora and Frank 2014
* Butyriboletus roseoflavus *	HKAS63593	Yunnan, SW China	KJ184559	KJ909517	KJ184571	–	Zhao et al. 2015
* Butyriboletus roseoflavus *	HKAS54099	Yunnan, SW China	KF739665	KJ909519	KF739779	–	Zhao et al. 2015
* Butyriboletus roseoflavus *	N.K. Zeng 2123 (FHMU 1387)	Yunnan, SW China	**MH879686**	**MH885348**	**MH879715**	–	this study
* Butyriboletus roseopurpureus *	E.E. Both3765	New York, USA	KT002617	KT002606	KT002641	–	Zhao et al. 2015
* Butyriboletus roseopurpureus *	JLF2566	West Virginia, USA	KC184467	KC184466	–	–	Arora and Frank 2014
* Butyriboletus roseopurpureus *	MB06-059	New York, USA	KF030262	KC184464	KF030410	–	Nuhn et al. 2013
* Butyriboletus sanicibus *	Arora99211	Yunnan, SW China	KC184470	KC184469	–	–	Arora and Frank 2014
*Butyriboletus* sp.	MHHNU7456	China	KT990539	–	KT990741	KT990378	Wu et al. 2016a
*Butyriboletus* sp.	HKAS52525	Yunnan, SW China	KF112337	–	KF112163	KF112671	Wu et al. 2014
*Butyriboletus* sp.	HKAS57774	Yunnan, SW China	KF112330	–	KF112155	KF112670	Wu et al. 2014
*Butyriboletus* sp.	HKAS59814	Hunan, central China	KF112336	–	KF112199	KF112699	Wu et al. 2014
*Butyriboletus* sp.	HKAS63528	Sichuan, SW China	KF112332	–	KF112156	KF112673	Wu et al. 2014
* Butyriboletus subappendiculatus *	MB000260	Germany	KT002618	KT002607	KT002642	–	Zhao et al. 2015
* Butyriboletus subsplendidus *	HKAS52661	Yunnan, SW China	KF112339	–	KF112169	KF112676	Wu et al. 2014
* Butyriboletus yicibus *	Arora9727	Yunnan, SW China	KC184475	KC184474	–	–	Arora and Frank 2014
* Butyriboletus yicibus *	HKAS57503	Yunnan, SW China	KT002620	KT002608	KT002644	–	Zhao et al. 2015
* Butyriboletus yicibus *	HKAS68010	Yunnan, SW China	KT002619	KJ909521	KT002643	–	Zhao et al. 2015
* Caloboletus calopus *	Bc1	Bavaria, Germany	AF456833	DQ679806	JQ327019	–	Zhao et al. 2014a
* Caloboletus calopus *	BR5020159063805	Montenau, Belgium	KJ184554	KJ605655	KJ184566	–	Zhao et al. 2014a
* Caloboletus calopus *	112606	California, USA	KF030279	–	–	–	Nuhn et al. 2013
* Caloboletus firmus *	MB06-060	New York, USA	KF030368	–	KF030408	–	Nuhn et al. 2013
* Caloboletus firmus *	NY00796115	Cayo, Belize	KJ605678	KJ605656	KJ619464	–	Zhao et al. 2014a
* Caloboletus guanyui *	N.K. Zeng 3058 (FHMU 2019)	Hainan, southern China	**MH879708**	**MH885365**	**MH879734**	**MH879751**	this study
* Caloboletus guanyui *	N.K. Zeng 3079 (FHMU 2040)	Hainan, southern China	**MH879709**	**MH885366**	**MH879736**	**MH879752**	this study
* Caloboletus guanyui *	N.K. Zeng 3257 (FHMU 2218)	Fujian, SE China	**MH879705**	–	**MH879732**	**MH879748**	this study
* Caloboletus guanyui *	N.K. Zeng 3261 (FHMU 2222)	Fujian, SE China	**MH879706**	–	**MH879733**	**MH879749**	this study
* Caloboletus guanyui *	N.K. Zeng 3263 (FHMU 2224)	Fujian, SE China	**MH879707**	**MH885364**	**MH879735**	**MH879750**	this study
* Caloboletus guanyui *	N.K. Zeng 3344 (FHMU 2809)	Hainan, southern China	–	–	**MK061357**	–	this study
* Caloboletus inedulis *	MB06-044	New York, USA	JQ327013	–	JQ327020	–	Halling et al. 2012
* Caloboletus inedulis *	HKAS80478	Florida, USA	KJ605671	KJ605657	KJ619465	–	Zhao et al. 2014a
* Caloboletus panniformis *	HKAS56164	Yunnan, SW China	KJ605674	KJ605667	KJ619466	–	Zhao et al. 2014a
* Caloboletus panniformis *	HKAS57410	Yunnan, SW China	KJ184555	KJ605659	KJ184567	–	Zhao et al. 2014a
* Caloboletus panniformis *	HKAS77530	Yunnan, SW China	KJ605670	KJ605661	KJ619470	–	Zhao et al. 2014a
* Caloboletus polygonius *	K(M)60247	Greece	KU317763	KU317753	–	–	GenBank
* Caloboletus radicans *	HKAS80856	France	KJ184557	KJ605662	KJ184569	–	Zhao et al. 2014a
*Caloboletus* sp.	HKAS53353	China	KF112410	–	KF112188	KF112668	Wu et al. 2014
* Caloboletus taienus *	GDGM44081	Guangdong, southern China	KY800414	KY800420	–	–	Zhang et al. 2017
* Caloboletus xiangtoushanensis *	GDGM44725	Guangdong, southern China	KY800416	KY800422	–	–	Zhang et al. 2017
* Caloboletus xiangtoushanensis *	GDGM44833	Guangdong, southern China	KY800415	KY800421	KY800418	–	Zhang et al. 2017
* Caloboletus xiangtoushanensis *	GDGM45160	Guangdong, southern China	KY800417	KY800423	KY800419	–	Zhang et al. 2017
* Caloboletus xiangtoushanensis *	N.K. Zeng 1330 (FHMU 883)	Fujian, SE China	**MH879702**	–	–	–	this study
* Caloboletus xiangtoushanensis *	N.K. Zeng 1331 (FHMU 884)	Fujian, SE China	**MH879703**	**MH885362**	–	–	this study
* Caloboletus xiangtoushanensis *	N.K. Zeng 1354 (FHMU 906)	Fujian, SE China	**MH879704**	**MH885363**	–	–	this study
* Caloboletus yunnanensis *	HKAS69214	Yunnan, SW China	KJ184556	KJ605663	KJ184568	–	Zhao et al. 2014a
* Caloboletus yunnanensis *	HKAS58694	Yunnan, SW China	KJ605672	KJ605664	KJ619470	–	Zhao et al. 2014a
* Chalciporus radiatus *	N.K. Zeng 1379 (FHMU 930)	Fujian, SE China	**MH879710**	**MH885367**	**MH879738**	–	this study
* Chalciporus radiatus *	N.K. Zeng 1414 (FHMU 959)	Fujian, SE China	**MH879711**	–	**MH879739**	–	this study
* Chalciporus radiatus *	N.K. Zeng 1808 (FHMU 2494)	Hainan, southern China	–	–	**MH879737**	–	this study
* Costatisporus cyanescens *	Henkel9067	Guyana	LC053662	LC054831	–	–	Smith et al. 2015
* Crocinoboletus laetissimus *	HKAS50232	Yunnan, SW China	KT990567	–	KT990762	–	Wu et al. 2016a
* Crocinoboletus rufoaureus *	HKAS53424	Hunan, central China	KF112435	–	KF112206	KF112710	Wu et al. 2014
* Cyanoboletus brunneoruber *	HKAS63504	Yunnan, SW China	KF112368	–	KF112194	–	Wu et al. 2014
* Cyanoboletus brunneoruber *	HKAS80579-1	Yunnan, SW China	KT990568	–	KT990763	–	Wu et al. 2016a
* Cyanoboletus brunneoruber *	HKAS80579-2	Yunnan, SW China	KT990569	–	KT990764	–	Wu et al. 2016a
* Cyanoboletus hymenoglutinosus *	DC14-010	India	KT860060	KT907355	–	–	Li et al. 2016
* Cyanoboletus instabilis *	HKAS59554	Yunnan, SW China	KF112412	–	KF112186	–	Wu et al. 2014
* Cyanoboletus instabilis *	FHMU1839	Yunnan, SW China	MG030466	MG030473	MG030478	–	Chai et al. 2018
* Cyanoboletus pulverulentus *	9606	USA	KF030313	–	KF030418	–	Nuhn et al. 2013
* Cyanoboletus pulverulentus *	RW109	Belgium	–	–	KT824046	–	Raspe et al. 2016
* Cyanoboletus pulverulentus *	MG126a	Italy	KT157062	KT157053	–	–	Gelardi et al. 2015
* Cyanoboletus pulverulentus *	MG456a	Azores Islands, Portugal	KT157063	KT157054	–	–	Gelardi et al. 2015
* Cyanoboletus pulverulentus *	MG628a	Italy	KT157064	KT157055	KT157073	–	Gelardi et al. 2015
* Cyanoboletus sinopulverulentus *	HKAS59609	Yunnan, SW China	KF112366	–	KF112193	–	Wu et al. 2014
*Cyanoboletus* sp.	HKAS76850	Hainan, southern China	KF112343	–	KF112187	–	Wu et al. 2014
*Cyanoboletus* sp.	HKAS52639	Yunnan, SW China	KF112367	–	KF112195	–	Wu et al. 2014
*Cyanoboletus* sp.	HKAS52601	Yunnan, SW China	KF112469	–	–	–	Wu et al. 2014
*Cyanoboletus* sp.	HKAS50292	Yunnan, SW China	KF112470	–	–	–	Wu et al. 2014
*Cyanoboletus* sp.	HKAS59418	China	KT990570	–	KT990765	–	Wu et al. 2016a
*Cyanoboletus* sp.	HKAS90208-1	China	KT990571	–	KT990766	–	Wu et al. 2016a
*Cyanoboletus* sp.	HKAS90208-2	China	–	–	KT990767	–	Wu et al. 2016a
*Cyanoboletus* sp.	PRM944518	USA	MF373585	–	–	–	Braeuer et al. 2018
* Exsudoporus frostii *	SAT1221511	Tennessee, USA	KP055021	–	KP055018	KP055027	Zhao et al. 2014b
* Exsudoporus frostii *	TENN067311	Tennessee, USA	KT002612	KT002601	KT002636	–	Zhao et al. 2015
* Lanmaoa angustispora *	HKAS74765	Yunnan, SW China	KF112322	–	KF112159	–	Wu et al. 2014
* Lanmaoa angustispora *	HKAS74752	Yunnan, SW China	KM605139	–	KM605154	–	Wu et al. 2016b
* Lanmaoa angustispora *	HKAS74759	Yunnan, SW China	KM605140	–	KM605155	–	Wu et al. 2016b
* Lanmaoa asiatica *	HKAS54094	Yunnan, SW China	KF112353	–	KF112161	–	Wu et al. 2014
* Lanmaoa asiatica *	HKAS63516	Yunnan, SW China	KT990584	–	KT990780	–	Wu et al. 2016a
* Lanmaoa asiatica *	HKAS63603	Yunnan, SW China	KM605142	–	KM605153	–	Wu et al. 2016b
* Lanmaoa asiatica *	FHMU1389	Yunnan, SW China	MG030470	MG030477	MG030481	–	Chai et al. 2018
* Lanmaoa asiatica *	FHMU1775	Yunnan, SW China	MG030469	–	MG030480	–	Chai et al. 2018
* Lanmaoa flavorubra *	NY775777	Costa Rica	JQ924339	–	KF112160	–	Wu et al. 2014
* Lanmaoa macrocarpa *	N.K. Zeng 3021 (FHMU 1982)	Hainan, southern China	**MH879684**	–	**MH879713**	–	this study
* Lanmaoa macrocarpa *	N.K. Zeng 3251 (FHMU 2212)	Fujian, SE China	**MH879685**	**MH885347**	**MH879714**	–	this study
* Lanmaoa pseudosensibilis *	DS615-07	USA	KF030257	–	KF030407	–	Nuhn et al. 2013
* Lanmaoa rubriceps *	FHMU 1756	Hainan, southern China	MG030465	MG030472	–	–	Chai et al. 2018
* Lanmaoa rubriceps *	FHMU 1757	Hainan, southern China	MG030467	MG030474	–	–	Chai et al. 2018
* Lanmaoa rubriceps *	FHMU 1763	Hainan, southern China	MG030468	MG030475	MG030479	–	Chai et al. 2018
* Lanmaoa rubriceps *	FHMU 2801	Hainan, southern China	MG030471	MG030476	–	–	Chai et al. 2018
* Lanmaoa rubriceps *	N.K. Zeng 3006 (FHMU 1967)	Hainan, southern China	**MH879683**	**MH885346**	**MH879712**	–	this study
*Lanmaoa* sp.	HKAS52518	Yunnan, SW China	KF112354	–	KF112162	–	Wu et al. 2014
* Neoboletus brunneissimus *	HKAS52660	Yunnan, SW China	KF112314	–	KF112143	KF112650	Wu et al. 2014
* Neoboletus ferrugineus *	HKAS77617	Guangdong, southern China	KT990595	–	KT990788	KT990430	Wu et al. 2016a
* Neoboletus ferrugineus *	HKAS77718	Guangdong, southern China	KT990596	–	KT990789	KT990431	Wu et al. 2016a
* Neoboletus flavidus *	HKAS58724	Yunnan, SW China	KU974140	–	KU974137	KU974145	Wu et al. 2016a
* Neoboletus flavidus *	HKAS59443	Yunnan, SW China	KU974139	–	KU974136	KU974144	Wu et al. 2016a
* Neoboletus hainanensis *	HKAS59469	Yunnan, SW China	KF112359	–	KF112175	KF112669	Wu et al. 2016a
* Neoboletus hainanensis *	HKAS90209	Hainan, southern China	KT990615	–	KT990809	KT990450	Wu et al. 2016a
* Neoboletus hainanensis *	HKAS63515	Yunnan, SW China	KT990614	–	KT990808	KT990449	Wu et al. 2016a
* Neoboletus hainanensis *	HKAS74880	Yunnan, SW China	KT990597	–	KT990790	KT990432	Wu et al. 2016a
* Neoboletus hainanensis *	N.K. Zeng 2128 (FHMU 1392)	Yunnan, SW China	**MH879690**	–	**MH879719**	–	this study
* Neoboletus luridiformis *	AT2001087	Berkshire, England	JQ326995	–	JQ327023	–	Halling et al. 2012
* Neoboletus magnificus *	HKAS54096	Yunnan, SW China	KF112324	–	KF112149	KF112654	Wu et al. 2014
* Neoboletus magnificus *	HKAS74939	Yunnan, SW China	KF112320	–	KF112148	KF112653	Wu et al. 2014
* Neoboletus multipunctatus *	HKAS76851	Hainan, southern China	KF112321	–	KF112144	KF112651	Wu et al. 2014
* Neoboletus multipunctatus *	N.K. Zeng 2498 (FHMU 1620)	Hainan, southern China	**MH879693**	**MH885354**	**MH879722**	–	this study
* Neoboletus multipunctatus *	N.K. Zeng3324 (FHMU 2808)	Hainan, southern China	**MK061360**	**MK061359**	**MK061358**	–	this study
* Neoboletus obscureumbrinus *	HKAS63498	Yunnan, SW China	KT990598	–	KT990791	KT990433	Wu et al. 2016a
* Neoboletus obscureumbrinus *	HKAS89027	Yunnan, SW China	KT990600	–	KT990794	KT990436	Wu et al. 2016a
* Neoboletus obscureumbrinus *	N.K. Zeng 3091 (FHMU 2052)	Hainan, southern China	**MH879694**	**MH885355**	**MH879723**	**MH879742**	this study
* Neoboletus obscureumbrinus *	N.K. Zeng 3094 (FHMU 2055)	Hainan, southern China	**MH879695**	**MH885356**	**MH879724**	**MH879743**	this study
* Neoboletus obscureumbrinus *	N.K. Zeng 3098 (FHMU 2059)	Hainan, southern China	**MH879696**	**MH885357**	**MH879725**	**MH879744**	this study
* Neoboletus rubriporus *	HKAS83026	Yunnan, SW China	KT990601	–	KT990795	KT990437	Wu et al. 2016a
* Neoboletus rubriporus *	HKAS89174	Yunnan, SW China	KT990602	–	KT990796	KT990438	Wu et al. 2016a
* Neoboletus rubriporus *	HKAS89181	Yunnan, SW China	KT990603	–	KT990797	–	Wu et al. 2016a
* Neoboletus rubriporus *	HKAS90210	Yunnan, SW China	KT990604	–	KT990798	KT990439	Wu et al. 2016a
* Neoboletus rubriporus *	MHKMU-L.P. Tang 1958	Yunnan, SW China	–	**MH885358**	**MH879726**	–	this study
* Neoboletus sanguineoides *	HKAS55440	Yunnan, SW China	KF112315	–	KF112145	KF112652	Wu et al. 2014
* Neoboletus sanguineoides *	HKAS57766	Yunnan, SW China	KT990605	–	KT990799	KT990440	Wu et al. 2016a
* Neoboletus sanguineoides *	HKAS63530	Sichuan, SW China	KT990607	–	KT990801	–	Wu et al. 2016a
* Neoboletus sanguineoides *	HKAS80823	Yunnan, SW China	KT990605	–	KT990799	KT990440	Wu et al. 2016a
* Neoboletus sanguineus *	HKAS80849	Yunnan, SW China	KT990609	–	KT990803	KT990443	Wu et al. 2016a
* Neoboletus sanguineus *	HKAS90211	Xizang, SW China	KT990610	–	KT990804	KT990444	Wu et al. 2016a
* Neoboletus sanguineus *	HKAS68587	Yunnan, SW China	KF112329	–	KF112150	KF112657	Wu et al. 2014
*Neoboletus* sp.	CMU58-ST-0237	–	KX017292	KX017301	–	–	GenBank
*Neoboletus* sp.	HKAS76851	Hainan, southern China	KF112321	–	KF112144	KF112651	Wu et al. 2014
*Neoboletus* sp.	HKAS50351	Yunnan, SW China	KF112318	–	–	KF112658	Wu et al. 2014
*Neoboletus* sp.	HKAS76660	Henan, Central China	KF112328	–	KF112180	KF112731	Wu et al. 2014
* Neoboletus thibetanus *	HKAS57093	Xizang, China	KF112326	–	–	KF112655	Wu et al. 2014
* Neoboletus tomentulosus *	HKAS53369	Fujian, SE China	KF112323	–	KF112154	KF112659	Wu et al. 2014
* Neoboletus tomentulosus *	HKAS77656	Guangdong, southern China	KT990611	–	KT990806	KT990446	Wu et al. 2016a
* Neoboletus tomentulosus *	N.K. Zeng 1285 (FHMU 841)	Fujian, SE China	**MH879691**	**MH885352**	**MH879720**	–	this study
* Neoboletus tomentulosus *	N.K. Zeng 1286 (FHMU 842)	Fujian, SE China	**MH879692**	**MH885353**	**MH879721**	–	this study
* Neoboletus venenatus *	HKAS57489	Yunnan, SW China	KF112325	–	KF112158	KF112665	Wu et al. 2014
* Neoboletus venenatus *	HKAS63535	Sichuan, SW China	KT990613	–	KT990807	KT990448	Wu et al. 2016a
* Rugiboletus brunneiporus *	HKAS68586	Xizang, SW China	KF112402	–	KF112197	–	Wu et al. 2014
* Rugiboletus brunneiporus *	HKAS83009	Xizang, SW China	KM605133	–	KM605146	–	Wu et al. 2016b
* Rugiboletus extremiorientalis *	HKAS76663	Henan, Central China	KM605135	–	KM605147	KM605170	Wu et al. 2016b
* Rugiboletus extremiorientalis *	HKAS74754	China	KT990639	–	KT990832	KT990469	Wu et al. 2016a
* Rubroboletus latisporus *	HKAS63517	Yunnan, SW China	KP055022	–	KP055019	KP055028	Zhao et al. 2014b
* Rubroboletus latisporus *	HKAS80358	Chongqing, SW China	KP055023	–	KP055020	KP055029	Zhao et al. 2014b
* Rubroboletus sinicus *	HKAS68620	Yunnan, SW China	KF112319	–	KF112146	KF112661	Zhao et al. 2014b
Sutorius aff. eximius	HKAS56291	Yunnan, SW China	KF112400	–	KF112208	KF112803	Wu et al. 2014
Sutorius aff. eximius	MHKMU-S.D. Yang 010	Yunnan, SW China	**MH879697**	**MH885359**	**MH879727**	–	this study
* Sutorius australiensis *	REH9280	Australia	JQ327031	–	JQ327031	–	Arora and Frank 2014
* Sutorius australiensis *	REH9441	Australia	JQ327006	–	JQ327032	MG212652	Halling et al. 2012
* Sutorius eximius *	REH9400	USA	JQ327004	–	JQ327029	–	Arora and Frank 2014
* Sutorius eximius *	HKAS52672	Yunnan, SW China	KF112399	–	KF112207	KF112802	Wu et al. 2014
* Sutorius eximius *	HKAS50420	Yunnan, SW China	KT990549	–	KT990750	KT990387	Wu et al. 2016a
* Sutorius eximius *	HKAS59657	China	KT990707	–	KT990887	KT990505	Wu et al. 2016a
* Sutorius eximius *	8594	Costa Rica	JQ327008	–	JQ327027	–	Halling et al. 2012
* Sutorius eximius *	995	Costa Rica	JQ327010	–	JQ327030	–	Halling et al. 2012
* Sutorius eximius *	986	Costa Rica	JQ327009	–	JQ327028	–	Halling et al. 2012
* Sutorius eximius *	8069	Indonesia	JQ327003	–	–	–	Halling et al. 2012
*Sutorius* sp.	N.K. Zeng 3297 (FHMU 2258)	Fujian, SE China	**MH879701**	–	**MH879731**	–	this study
*Sutorius* sp.	ECV3603	Thailand	JQ327000	–	JQ327033	–	Halling et al. 2012
*Sutorius* sp.	01-528	Zambia	JQ327002	–	–	–	Halling et al. 2012
* Sutorius subrufus *	N.K. Zeng 3043 (FHMU 2004)	Hainan, southern China	**MH879698**	**MH885360**	**MH879728**	**MH879745**	this study
* Sutorius subrufus *	N.K. Zeng 3045 (FHMU 2006)	Hainan, southern China	**MH879699**	**MH885361**	**MH879729**	**MH879746**	this study
* Sutorius subrufus *	N.K. Zeng 3140 (FHMU 2101)	Hainan, southern China	**MH879700**	–	**MH879730**	**MH879747**	this study

### Dataset assembly

For the concatenated multilocus dataset of *Butyriboletus*, 14 sequences (four of 28S, four of ITS, four of *tef1*, and two of *rpb*2) from four collections were newly generated (Table 1) and then combined with selected sequences from previous studies (Table 1). *Rugiboletusextremiorientalis* (Lj.N. Vassiljeva) G. Wu & Zhu L. Yang was chosen as outgroup on the basis of the phylogeny in Wu et al. (2016a). For the concatenated multilocus dataset of *Caloboletus*, *Neoboletus*, and *Sutorius*, 68 sequences (21 of 28S, 16 of ITS, 20 of *tef1*, 11 of *rpb*2) from 23 collections were newly generated and deposited in GenBank (Table 1) and then combined with selected sequences from previous studies (Table 1). *Crocinoboletuslaetissimus* (Hongo) N.K. Zeng et al. and *Cr.rufoaureus* (Massee) N.K. Zeng et al. were chosen as outgroup based on the phylogeny in Wu et al. (2016a). For the concatenated multilocus dataset of *Lanmaoa*, eight sequences (three of 28S, two of ITS, and three of *tef1*) from three collections were newly generated and deposited in GenBank (Table 1), and then combined with selected sequences from previous studies (Table 1). *Rugiboletusbrunneiporus* G. Wu & Zhu L. Yang was chosen as outgroup on the basis of the phylogeny in Wu et al. (2016a). To test for phylogenetic conflict among the different genes in three combined datasets (*Butyriboletus*, *Caloboletus* + *Neoboletus* + *Sutorius*, *Lanmaoa*), the partition homogeneity (PH) or incongruence length difference (ILD) test was performed with 1000 randomized replicates, using heuristic searches with simple addition of sequences in PAUP* 4.0b10 (Swofford 2002). The results of the partition homogeneity test showed that the phylogenetic signals present in the different gene fragments were not in conflict. Then the sequences of different genes in three combined datasets (*Butyriboletus*, *Caloboletus* + *Neoboletus* + *Sutorius*, *Lanmaoa*) were aligned with MAFFT v. 6.8 using algorithm E-INS-i (Katoh et al. 2005) and manually optimized on BioEdit v. 7.0.9 (Hall 1999). The sequences of the different genes were concatenated in three combined datasets (*Butyriboletus*, *Caloboletus* + *Neoboletus* + *Sutorius*, *Lanmaoa*) using Phyutility v. 2.2 for further analyses (Smith and Dunn 2008).

### Phylogenetic analyses

The three combined datasets (*Butyriboletus*, *Caloboletus* + *Neoboletus* + *Sutorius*, *Lanmaoa*) were all analyzed by using maximum likelihood (ML) and Bayesian inference (BI). Maximum likelihood tree generation and bootstrap analyses were performed with the program RAxML 7.2.6 (Stamatakis 2006) running 1000 replicates combined with an ML search. Bayesian analysis with MrBayes 3.1 (Huelsenbeck and Ronquist 2005) implementing the Markov Chain Monte Carlo (MCMC) technique and parameters predetermined with MrModeltest 2.3 (Nylander 2004) was performed. The model of evolution used in the Bayesian analysis was determined with MrModeltest 2.3 (Nylander 2004). For the combined dataset of *Butyriboletus*, the best-fit likelihood models of 28S, ITS1+ITS2, 5.8S, *tef1* and *rpb*2 were GTR+I+G, HKY+I+G, K80, SYM+I+G and K80+I+G, respectively; for the combined dataset of *Caloboletus*, *Neoboletus*, and *Sutorius*, the best-fit likelihood models of 28S, ITS1+ITS2, 5.8S, *tef1* and *rpb*2 were GTR+I+G, HKY+I+G, K80, SYM+I+G and SYM+I+G, respectively; for the combined dataset of *Lanmaoa*, the best-fit likelihood models of 28S, ITS1+ITS2, 5.8S and *tef1* were GTR+I+G, GTR+I, K80 and SYM+G, respectively. Bayesian analysis was run with one cold and three heated chains and sampled every 100 generations; trees sampled from the first 25% of the generations were discarded as burn-in; the average standard deviation of split frequencies was restricted to be below 0.01, and Bayesian posterior probabilities (PP) were then calculated for a majority consensus tree of the retained Bayesian trees.

## Results

### Molecular data

**Figure 1.**
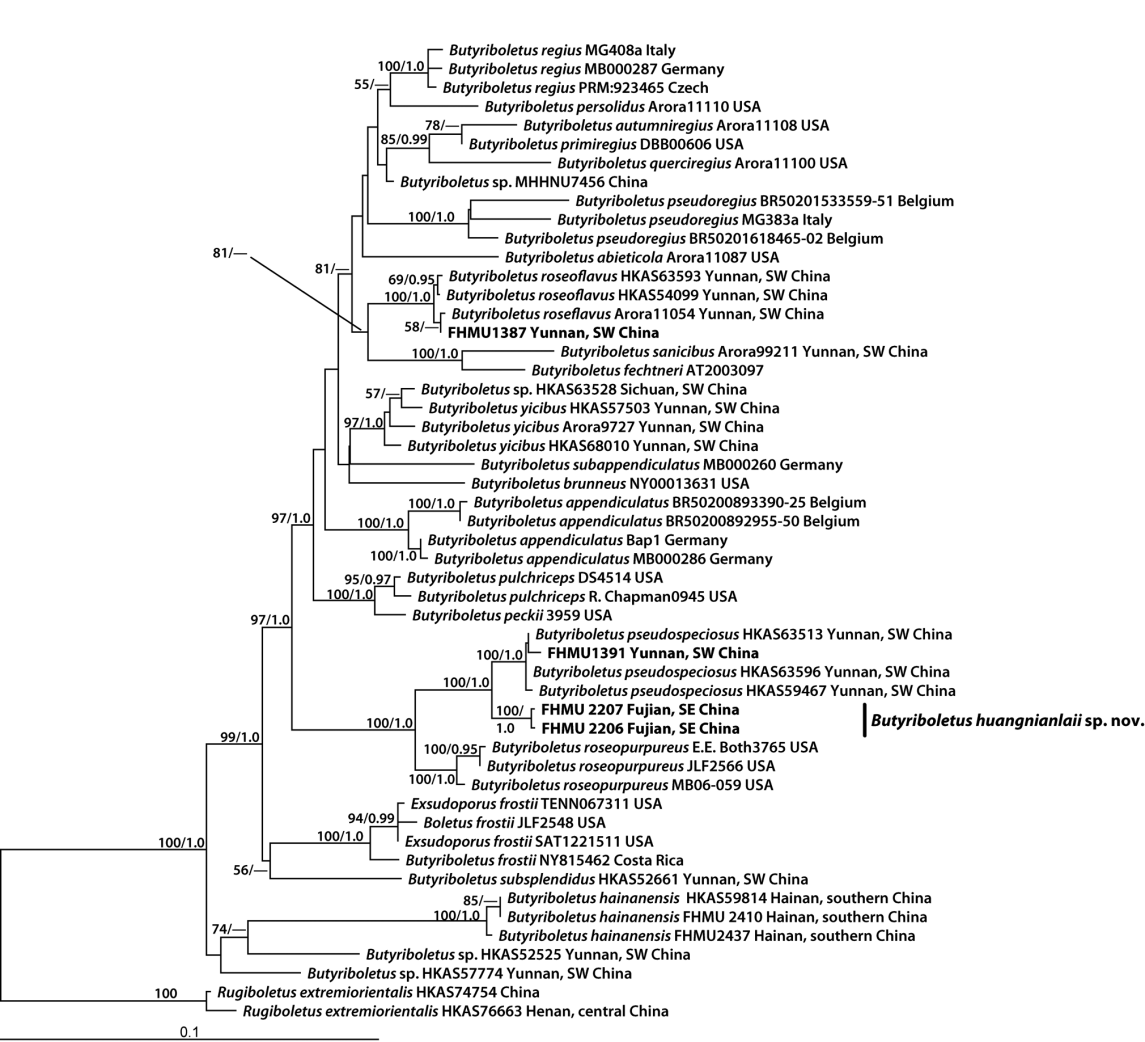
Phylogenetic placement of *Butyriboletushuangnianlaii* inferred from a multilocus (28S, ITS, *tef1*, *rpb*2) dataset using RAxML. BS ≥ 50% and PP ≥ 0.95 are indicated above or below the branches as RAxML BS/PP.

The four-locus dataset (28S + ITS + *tef1* + *rpb*2) of *Butyriboletus* consisted of 52 taxa and 3116 nucleotide sites (Fig. 1). The aligned dataset was submitted to TreeBASE (http://purl.org/phylo/treebase/phylows/study/TB2:S23508). The molecular phylogenetic analyses showed that the collections numbered as FHMU 2206 and FHMU 2207 respectively grouped together with a high statistical support (BS = 100, PP = 1), forming an independent lineage within *Butyriboletus* (Fig. 1).

**Figure 2.**
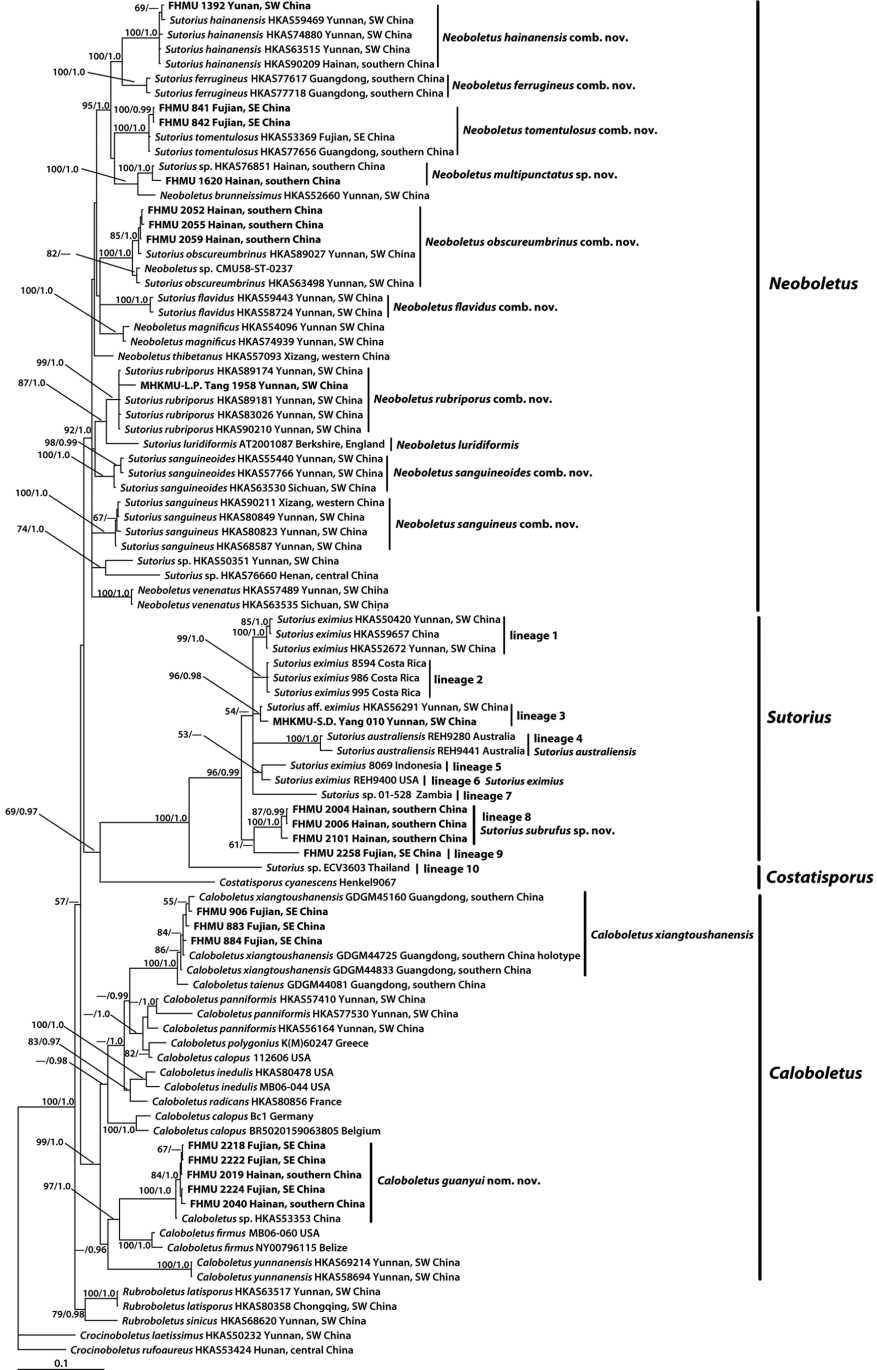
Phylogenetic placement of *Neoboletusmultipunctatus*, *Sutoriussubrufus* and *Caloboletusguanyui* inferred from a multilocus (28S, ITS, *tef1*, *rpb*2) dataset using RAxML. BS ≥ 50% and PP ≥ 0.95 are indicated above or below the branches as RAxML BS/PP.

The four-locus dataset (28S + ITS + *tef1* + *rpb*2) with *Caloboletus*, *Neoboletus*, and *Sutorius* consisted of 93 taxa and 3228 nucleotide sites (Fig. 2). The aligned dataset was submitted to TreeBASE (http://purl.org/phylo/treebase/phylows/study/TB2:S23509). The molecular phylogenetic analyses indicated each of the previously described genera, viz. *Neoboletus*, *Sutorius*, *Costatisporus*T.W. Henkel & M.E. Sm., and *Caloboletus*, forms an independent clade with a high statistical support respectively (Fig. 2). In the genus *Neoboletus*, one collection numbered as FHMU 1392 and one previously described *S.hainanensis* (T.H. Li & M. Zang) G. Wu and Zhu L. Yang grouped together with a strong statistical support (BS = 100, PP = 1), forming an independent lineage; two collections numbered as FHMU 841 and FHMU 842 respectively and one previously described *S.tomentulosus* (M. Zang et al.) G. Wu & Zhu L. Yang grouped together with a high statistical support (BS = 100, PP = 1), forming an independent lineage; one collection tentatively named *Sutorius* sp. (HKAS 76851) in a previous study (Wu et al. 2016a) and one specimen numbered as FHMU 1620 grouped together with a high statistical support (BS = 100, PP = 1), forming an independent lineage; three specimens numbered as FHMU 2052, FHMU 2055, FHMU 2059 respectively and one previously described *S.obscureumbrinus* (Hongo) G. Wu & Zhu L. Yang grouped together with a high statistical support (BS = 100, PP = 1), forming an independent lineage (Fig. 2). In the genus *Sutorius*, the specimens numbered as FHMU 2004, FHMU 2006 and FHMU 2101 respectively grouped together with a high statistical support (BS = 100, PP = 1), forming an independent lineage (Fig. 2). In the genus *Caloboletus*, the materials numbered as FHMU 883, FHMU 884, FHMU 906 respectively and the holotype of *C.xiangtoushanensis* Ming Zhang et al. grouped together with a high statistical support (BS = 100, PP = 1), forming an independent lineage; the collections numbered as FHMU 2019, FHMU 2040, FHMU 2218, FHMU 2222 and FHMU 2224 respectively grouped together with a strong statistical support (BS = 100, PP = 1), forming an independent lineage (Fig. 2).

**Figure 3.**
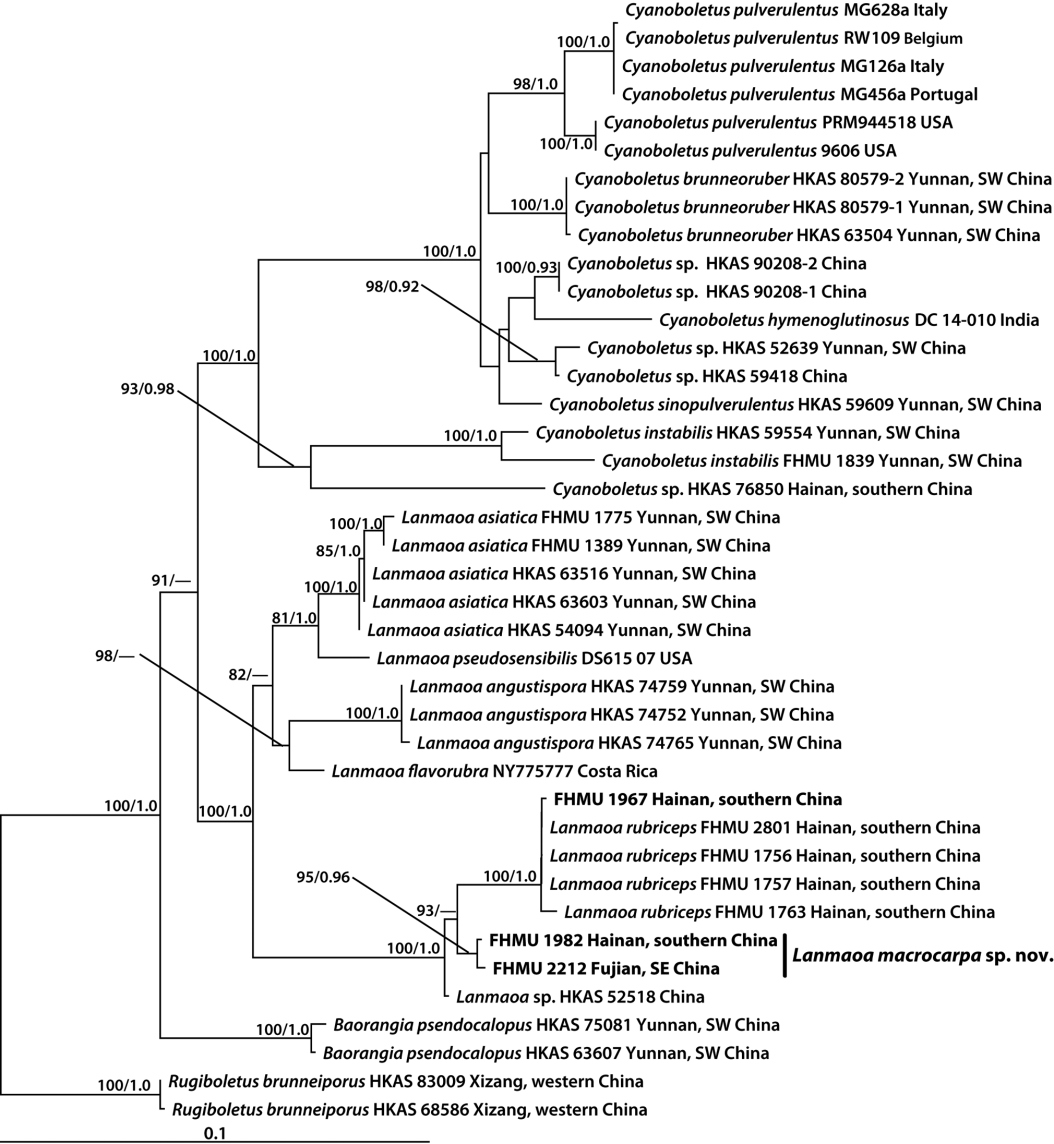
Phylogenetic placement of *Lanmaoamacrocarpa* inferred from a multilocus (28S, ITS, *tef1*) dataset using RAxML. BS ≥ 50% and PP ≥ 0.95 are indicated above or below the branches as RAxML BS/PP.

The three-locus dataset (28S + ITS + *tef1*) of *Lanmaoa* consisted of 40 taxa and 2007 nucleotide sites (Fig. 3). The aligned dataset was submitted to TreeBASE (http://purl.org/phylo/treebase/phylows/study/TB2:S23510). The molecular phylogenetic analyses showed that the collections numbered as FHMU 1982 and FHMU 2212 respectively grouped together with a high statistical support (BS = 100, PP = 1), forming an independent lineage within *Lanmaoa* (Fig. 3).

### Taxonomy

#### *Butyriboletus* D. Arora & J.L.Frank

*Butyriboletus*, typified by *But.appendiculatus* (Schaeff.) D. Arora & J.L.Frank, was erected to accommodate the “butter boletes”, which are mainly characterized by yellow hymenophore and context staining blue when injured and stipe surface usually covered with reticulations (Arora and Frank 2014; Zhao et al. 2015). Until now, six species, including *But.hainanensis* N.K. Zeng et al., *But.pseudospeciosus* Kuan Zhao & Zhu L.Yang, *But.roseoflavus* (Hai B.Li & Hai L.Wei) D.Arora & J.L.Frank, *But.sanicibus* D. Arora & J.L.Frank, *But.subsplendidus* (W.F. Chiu) Kuan Zhao et al., and *But.yicibus* D. Arora & J.L.Frank have been described from China (Arora and Frank 2014; Liang et al. 2016; Wu et al. 2016a). Herein, we describe another novel species.

##### 1. Butyriboletus huangnianlaii

Taxon classificationPlantaeBoletalesBoletaceae

N.K. Zeng, H. Chai & Zhi Q. Liang sp. nov.

MB828521

Figures 4a, b , 7

###### Typification.

CHINA. Fujian Province: Sanming City, Geshikao National Forest Park, elev. 420 m, 16 August 2017, *N.K. Zeng 3246* (FHMU 2207, holotype). GenBank accession numbers: 28S = MH879689, ITS = MH885351, *tef1* = MH879718, *rpb2* = MH879741.

###### Etymology.

Latin, “*huangnianlaii*” is named after Chinese mycologist Nian-Lai Huang, in honor of his contribution to mycology.

###### Description.

*Basidiomata* medium-sized to large. *Pileus* 5–11 cm in diameter, convex to applanate; surface dry, finely tomentose, pale brown (5D1–4D2), brown to reddish brown (5C2–6C2); context 0.6–2.2 cm thick in the center of the pileus, yellowish to yellow, changing blue quickly when injured. *Hymenophore* poroid, adnate or slightly depressed around apex of stipe; pores angular, about 0.5 mm in diameter, yellowish white (30A2) to yellowish brown (4A4), changing blue quickly when injured; tubes 0.4–0.8 cm in length. *Stipe* 4.5–8 × 1.3–2.5 cm, central, subcylindric, solid; surface dry, yellowish (30A2) when young, then brownish red (8D5), reticulate nearly to base; reticulum yellowish (1A2) when young, then brownish red (8D5); context yellowish to yellow, changing blue quickly when injured; basal mycelium white (1A1). *Odor* indistinct.

*Basidia* 20–31 × 6–9 μm, clavate, thin-walled, colorless to yellowish in KOH; four-spored, sterigmata 3–4 μm in length. *Basidiospores* [40/2/2] (7–)7.5–10.5(–11) × 3–4 μm, Q=(2.00–)2.14–2.86(–3.14), *Q_m_*=2.51 ± 0.27, subfusoid and inequilateral in side view with a weak or distinct suprahilar depression, elliptic-fusiform to subfusiform in ventral view, slightly thick-walled (to 0.5 μm), olive-brown to yellowish brown in KOH, smooth. *Hymenophoral trama* boletoid; composed of colorless to yellowish in KOH, 3–10 μm wide, thin- to slightly thick-walled (to 0.5 μm) hyphae. *Cheilocystidia* 32–53 × 7–12 μm, fusiform or subfusiform, thin-walled, yellowish in KOH, no encrustations. *Pleurocystidia* 40–60 × 8–13 μm, fusiform or subfusiform, thin-walled, yellowish in KOH, no encrustations. *Pileipellis* a trichoderm about 110 μm thick, composed of slightly interwoven, nearly colorless in KOH, 4–6 μm wide, thin-walled hyphae; terminal cells 30–50× 4–8 μm, clavate or subclavate, with obtuse apex. *Pileal trama* made up of hyphae 8–12 μm in diameter, thin-walled, colorless in KOH. *Stipitipellis* hymeniform about 120–140 μm thick, composed of thin- to slightly thick-walled (to 0.5 μm) emergent hyphae, colorless to yellowish in KOH, with clavate, subclavate, fusiform or subfusiform terminal cells (15–45 × 4–9 μm) , and occasionally with scattered clavate, 4-spored basidia. *Stipe trama* composed of longitudinally arranged, parallel hyphae 3.5–7 μm wide, cylindrical, thin- to slightly thick-walled (up to 0.5 μm), colorless to yellowish in KOH, parallel hyphae. *Clamp connections* absent in all tissues.

**Figure 4.**
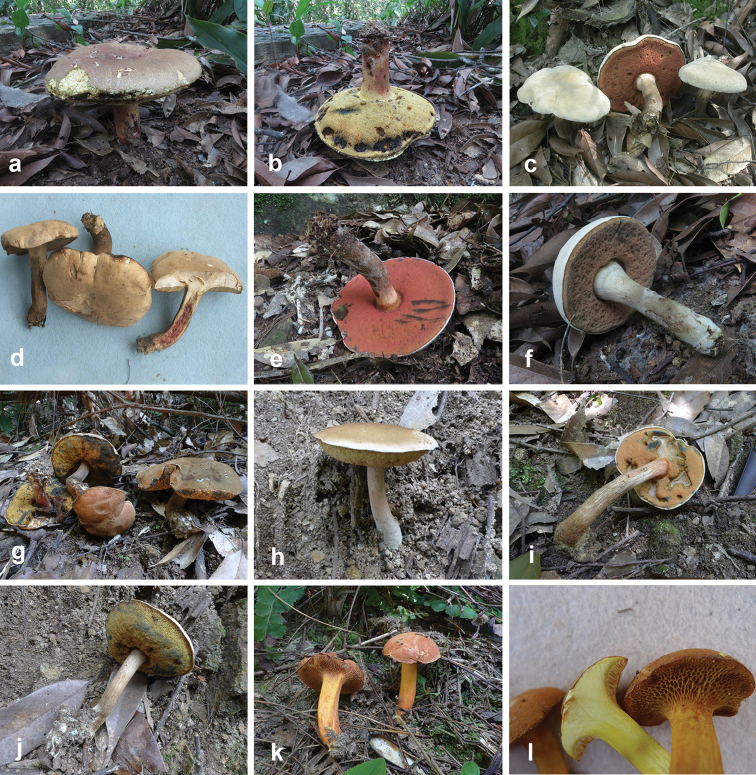
Basidiomata of boletes. **a, b***Butyriboletushuangnianlaii* (FHMU 2207, holotype) **c–f***Caloboletusguanyui* (**c–d** from FHMU 399; **e** from FHMU 2224; f from FHMU 2222) **g–j***Caloboletusxiangtoushanensis* (**g** from FHMU 883 **h, j** from FHMU 906 **i** from FHMU 884) **k, l***Chalciporusradiatus* (FHMU 930). Photos by N.K. Zeng.

###### Habitat.

Scattered on the ground in forests dominated by *Castanopsiskawakamii* Hay.

###### Distribution.

Southeastern China.

###### Additional specimens examined.

CHINA. Fujian Province: Sanming City, Geshikao National Forest Park, elev. 420 m, 16 August 2017, *N.K. Zeng 3245* (FHMU 2206).

###### Note.

*Butyriboletushuangnianlaii* is characterized by a medium-sized to large basidioma, pileal surface densely covered with pale brown to reddish brown squamules, smaller basidiospores, and its association with fagaceous trees. It is both morphologically similar and phylogenetically related to *But.pseudospeciosus* and *But.roseoflavus* (Fig. 1). However, *But.pseudospeciosus*, originally described from Yunnan Province of southwestern China, has a tomentose pileus without a reddish tinge, surface of pileus and stipe promptly staining blue when bruised, narrower cystidia and longer basidiospores measuring 9–11 × 3.5–4 μm (Wu et al. 2016a); *But.roseoflavus*, originally described from Zhejiang Province of southeastern China, has a pinkish to purplish red or rose-red pileus with tomentose surface, longer basidiospores measuring 9–12 × 3–4 μm, and its association with *Pinus* spp. (Arora and Frank 2014; Li et al. 2014; Wu et al. 2016a).

#### *Caloboletus* Vizzini

*Caloboletus*, typified by *C.calopus* (Pers.) Vizzini, is mainly characterized by yellow tubes, yellow or more rarely orange to red pores changing to blue when injured, bitter taste of the context due to the presence of calopin and cyclocalopin (Hellwig et al. 2002; Vizzini 2014; Zhao et al. 2014a; Wu et al. 2016a; Zhang et al. 2017). Until now, four species, including *C.panniformis* (Taneyama & Har. Takah.) Vizzini, *C.taienus* (W.F. Chiu) Ming Zhang and T.H. Li, *C.xiangtoushanensis* Ming Zhang et al., and *C.yunnanensis* Kuan Zhao & Zhu L. Yang, have been found in China (Zhao et al. 2014a; Wu et al. 2016a; Zhang et al. 2017). We describe two *Caloboletus* species here.

##### 2. Caloboletus guanyui

Taxon classificationPlantaeBoletalesBoletaceae

N.K. Zeng, H.Chai & S.Jiang nom. nov.

MB828522

Figures 4c–f , 8

Boletus quercinus Hongo, Memoirs of Shiga University 17: 92, 1967 (nom. illeg., later homonym)
non Boletusquercinus Schrad., Spicilegium Florae Germanicae 1: 157, 1794
non Boletusquercinus (Pilát) Hlaváček, Mykologický Sborník 67(3): 87, 1990 (nom. illeg., later homonym)

###### Etymology.

Latin, “*guanyui*” is named for Guan Yu, a historic Chinese hero, said to have a reddish face, and thus sharing the same color of pores of the species when young.

###### Description.

*Basidiomata* medium-sized to large. *Pileus* 5–10 cm in diameter, convex to applanate; surface dry, finely tomentose, dirty white to pale brown; context 0.5–1.8 cm thick in the center of the pileus, white, changing bluish quickly when injured, then back to white. *Hymenophore* poroid, depressed around apex of stipe; pores subround, 0.3–0.5 mm in diameter, reddish to reddish brown when young, then yellow or yellowish brown, changing bluish black when injured; tubes about 0.5–1 cm in length, yellowish, changing bluish quickly when injured. *Stipe* 5.5–9 × 0.7–1.5 cm, central, subcylindric, solid, usually flexuous; surface dry, densely covered with pale brown, brown to reddish brown, minute squamules; context white, sometimes tinged with pale red, unchanging in color when injured; basal mycelium white. *Odor* indistinct.

*Basidia* 21–30 × 6–8 μm, clavate, thin-walled, colorless to yellowish in KOH; four-spored, sterigmata 3–4 μm in length. *Basidiospores* [220/12/5] (8.5–)9–11(–12) × 3.5–4.5 μm, Q=(2.00–)2.22–2.67(–2.86), *Q_m_*=2.43 ± 0.17, subfusoid and inequilateral in side view with a weak or distinct suprahilar depression, elliptic-fusiform to subfusiform in ventral view, slightly thick-walled (to 0.5 μm), olive-brown to yellowish brown in KOH, smooth. *Hymenophoral trama* boletoid; composed of yellowish in KOH, 4–10 μm wide, thin-walled hyphae. *Cheilocystidia* 25–40 × 7–10 μm, fusiform or subfusiform, thin-walled, colorless to yellowish in KOH, no encrustations. *Pleurocystidia* 35–45 × 6–11 μm, fusiform or subfusiform, thin-walled, colorless to yellowish in KOH, no encrustations. *Pileipellis* a trichoderm about 100–200 μm thick, composed of slightly interwoven, nearly colorless in KOH, 5–8 μm wide, thin-walled hyphae; terminal cells 28–35 × 5–10 μm, clavate or subclavate, with obtuse apex. *Pileal trama* made up of hyphae 4–8 μm in diameter, slightly thick-walled (to 0.5 μm), colorless to yellowish in KOH. *Stipitipellis* hymeniform about 80–100 μm thick, composed of thin-walled emergent hyphae, yellowish in KOH, with clavate, subclavate, fusiform or subfusiform terminal cells (27–43 × 6–11 μm), and occasionally with scattered clavate, 4-spored basidia. *Stipe trama* composed of longitudinally arranged, parallel hyphae 3–6 μm wide, cylindrical, thin-walled, colorless to yellowish in KOH. *Clamp connections* absent in all tissues.

###### Habitat.

Gregarious on the ground in forests dominated by *Castanopsiskawakamii* Hay. or *Lithocarpus* spp.

###### Distribution.

Southeastern and southern China; Japan (Hongo 1967).

###### Specimens examined.

CHINA. Hainan Province: Ledong County, Yinggeling National Nature Reserve, elev. 650 m, 4 June 2017, *N.K. Zeng 3058* (FHMU 2019); same location, 5 June 2017, *N.K. Zeng 3079* (FHMU 2040). Fujian Province: Zhangping County, Tiantai National Forest Park, elev. 350 m, 28 August 2009, *N.K. Zeng 635* (FHMU 399); Sanming City, Geshikao National Forest Park, elev. 420 m, 16 August 2017, *N.K. Zeng 3257* (FHMU 2218); same location and date, *N.K. Zeng 3261* (FHMU 2222); Yongan City, Tianbaoyan National Nature Reserve, elev. 600 m, 17 August 2017, *N.K. Zeng 3263* (FHMU 2224).

###### Note.

*Caloboletusguanyui* was originally described as *B.quercinus* from Japan (Hongo 1967). Nomenclaturally, the epithet *quercinus* of this species is an illegitimate name, because Schrader (1794) described a species using the same epithet before Hongo (1967). Therefore, the new epithet *guanyui* is proposed here for this species. Moreover, morphological and molecular evidence indicates the taxon is a member of the genus *Caloboletus* (Fig. 2), and is characterized by a dirty-white to pale-brown pileus, pores reddish to reddish brown when young, then yellow or yellowish brown, changing bluish black when injured, and a stipe densely covered with pale-brown, brown to reddish-brown squamules. Morphologically, *C.taienus* and *C.xiangtoushanensis* also have reddish pores (Bessette et al. 2016; Zhang et al. 2017), however, a dirty-white to pale-brown pileus easily distinguishes *C.guanyui* from the two taxa. Phylogenetically *C.guanyui* is closely related to *C.firmus* (Frost) Vizzini (Fig. 2), however, *C.firmus* has a stipe covered with whitish or reddish reticula, and it is restricted to North and Central America (Bessette et al. 2016).

##### 3. Caloboletus xiangtoushanensis

Taxon classificationPlantaeBoletalesBoletaceae

Ming Zhang, T.H. Li & X.J. Zhong, Phytotaxa 309: 119, 2017

Figures 4g–j , 9

###### Description.

*Basidiomata* medium-sized to large. *Pileus* 5.5–11 cm in diameter, convex to plane; surface dry, tomentose, yellowish brown, pale brown to brown; context 1–1.5 cm thick in the center of the pileus, yellowish, changing blue quickly when injured. *Hymenophore* poroid, adnate to depressed around apex of stipe; pores subround to angular, 0.5–1 mm in diameter, yellow, sometimes brownish red, changing blue quickly when injured; tubes 0.5–1.4 cm in length, yellowish, changing blue quickly when injured. *Stipe* 5–9 × 0.9–1.6 cm, central, subcylindric, solid, usually flexuous; surface dry, upper part covered with reddish brown, minute squamules, middle and lower part covered with brown minute squamules; context yellowish, changing blue quickly when injured; basal mycelium white. *Odor* indistinct.

*Basidia* 25–35 × 5–10 μm, clavate, thin-walled, colorless to yellowish in KOH; four-spored, sterigmata 3–4 μm in length. *Basidiospores* [140/8/3] (9.5–)10–11.5(–13) × 3.5–4.5 μm, Q=(2.11–)2.44–3.00(–3.29), *Q_m_*=2.76 ± 0.21, subfusoid and inequilateral in side view with a weak or distinct suprahilar depression, elliptic-fusiform to subfusiform in ventral view, slightly thick-walled (to 0.5 μm), olive-brown to yellowish brown in KOH, smooth. *Hymenophoral trama* boletoid; composed of colorless to yellowish in KOH, 4–10 μm wide, thin-walled hyphae. *Cheilocystidia* 25–45 × 7–10 μm, fusiform or subfusiform, thin-walled, colorless in KOH, no encrustations. *Pleurocystidia* 30–50 × 7–12 μm, fusiform or subfusiform, thin-walled, colorless in KOH, no encrustations. *Pileipellis* a trichoderm about 70–100 μm thick, composed of slightly interwoven, colorless or yellowish in KOH, 4–7 μm wide, thin-walled hyphae; terminal cells 35–55 × 4–7 μm, clavate or subclavate, with obtuse apex. *Pileal trama* made up of hyphae 3.5–7 μm in diameter, thin-walled, colorless to yellowish in KOH. *Stipitipellis* hymeniform about 60–80 μm thick, composed of thin- to slightly thick-walled (to 0.5 μm) emergent hyphae, colorless to yellowish in KOH, with clavate, subclavate, fusiform or subfusiform terminal cells (15–46 × 5–8 μm), and occasionally with scattered clavate, four-spored basidia. *Stipe trama* composed of longitudinally arranged, parallel hyphae 3.5–8 μm wide, cylindrical, thin- to slightly thick-walled (to 0.5 μm), yellowish in KOH. *Clamp connections* absent in all tissues.

###### Habitat.

Solitary or gregarious on the ground in forests dominated by fagaceous trees.

###### Distribution.

Southeastern and southern China.

###### Specimens examined.

CHINA. Fujian Province: Zhangping County, Xinqiao Town, Chengkou Village, elev. 350 m, 30 July 2013, *N.K. Zeng 1330* (FHMU 883); same location and date, *N.K. Zeng 1331* (FHMU 884); same location, 1 August 2013, *N.K. Zeng 1354* (FHMU 906).

###### Notes.

Our recent collections and the holotype of *C.xiangtoushanensis*, a species originally described from Guangdong Province of southern China (Zhang et al. 2017), phylogenetically group together with a strong statistical support (Fig. 2), which indicates that these specimens should be recognized as *C.xiangtoushanensis*. It is new to Fujian Province. Morphologically, several features of our collections also match well with the protologue of *C.xiangtoushanensis* (Zhang et al. 2017), but reticulations on the stipe were not observed in our specimens. Moreover, pores of our specimens are sometimes brownish red. In appearance, *C.xiangtoushanensis* is highly similar to Japanese *B.bannaensis* Har. Takah., which needs further confirmation for generic placement (Takahashi 2007). However, *B.bannaensis* has rufescent and faintly cyanescent context, small basidiospores measuring 6.5–9 × 3.5–4 μm, and narrower cystidia (Takahashi 2007). The molecular analyses also indicates that *C.xiangtoushanensis* is closely related to *C.taienus* (W.F. Chiu) Ming Zhang and T.H. Li (Fig. 2), a species originally described from Yunnan Province (Chiu 1948); their morphological differences have been elucidated in a previous study (Zhang et al. 2017).

#### *Chalciporus* Bataille

*Chalciporus*, typified by *Ch.piperatus* (Bull.) Bataille, is an early branching lineage in the Boletaceae (Nuhn et al. 2013; Wu et al. 2014, 2016b) and is characterized by a pinkish-red to reddish-brown hymenophore. Several taxa, including *Ch.citrinoaurantius* Ming Zhang & T.H. Li, *Ch.hainanensis* Ming Zhang & T.H. Li, *Ch.radiatus* Ming Zhang & T.H. Li, and *Ch.rubinelloides* G.Wu & Zhu L. Yang, were recently described from China (Zhang et al. 2015, 2017; Wu et al. 2016b). Here, *Ch.radiatus* is redescribed based on new collections from subtropical and tropical China.

##### 4. Chalciporus radiatus

Taxon classificationPlantaeBoletalesBoletaceae

Ming Zhang & T.H. Li, Mycoscience 57: 21, 2016

Figures 4k, l , 10

###### Description.

*Basidiomata* small. *Pileus* 2.5–5 cm in diameter, subhemispherical to convex when young, then applanate; surface dry, pale yellowish brown, densely covered with pale yellowish-brown, yellowish-brown, brown to reddish-brown squamules; margin decurved; context 0.6–1 cm thick in the center of the pileus, yellowish, unchanging in color when injured. *Hymenophore* poroid, slightly decurrent; pores radially strongly elongated, yellow to pale yellowish brown, reddish with age, unchanging in color when injured; tubes 0.2–0.4 cm in length, yellowish, unchanging in color when injured. *Stipe* 2.5–4.5 × 0.5–1 cm, central, subcylindric, solid; surface dry, yellow, covered with yellowish brown, brown to reddish-brown squamules; context yellowish, unchanging in color when injured; annulus absent; basal mycelium yellow. *Odor* indistinct.

*Basidia* 23–34 × 7–10 μm, clavate, thin-walled, four-spored; sterigmata 5–6 μm in length. *Basidiospores* [101/5/4] 6–7(–8) × 3–4 μm, Q = (1.63–)1.71–2.14(–2.33), *Q_m_* = 1.91 ± 0.15, subfusoid and inequilateral in side view with a weak or distinct suprahilar depression, elliptic-fusiform to subfusiform in ventral view, slightly thick-walled (to 0.5 μm), olive-brown to yellowish brown in KOH, smooth. *Hymenophoral trama* boletoid. *Cheilocystidia* 57–75 × 8–10 μm, abundant, subfusiform or fusiform, thin-walled, with pale yellowish-brown to yellowish-brown contents, without encrustations. *Pleurocystidia* 60–76 × 7–9 μm, abundant, fusiform or subfusiform, thin-walled, with pale yellowish-brown to yellowish-brown contents, without encrustations. *Pileipellis* a trichoderm 200–230 μm thick, composed of rather vertically arranged, sometimes slightly interwoven, pale yellowish-brown to yellowish-brown in KOH, thin-walled hyphae 4–10 μm in diameter; terminal cells 25–50 × 6–9 μm, narrowly clavate or subcylindrical, with obtuse apex. *Pileal trama* composed of thin- to slightly thick-walled (up to 0.5μm) hyphae 2–8 μm in diameter. *Stipitipellis* hymeniform composed of thin- walled hyphae with clavate, subclavate, subfusiform or fusiform terminal cells (13–80 × 5–9 μm). *Stipe trama* composed of cylindrical, thin- to slightly thick-walled (to 0.5 μm) parallel hyphae 5–11 μm in diameter. *Clamp connections* absent in all tissues.

###### Habitat.

Solitary, scattered or gregarious on the ground in forests of *Pinusmassoniana* Lamb. or *P.latteri* Mason.

###### Distribution.

Central (Zhang et al. 2015), southeastern, and southern China.

###### Specimens examined.

CHINA. Fujian Province: Zhangping County, Xinqiao Town, Chengkou Village, elev. 370 m, 4 August 2013, *N.K. Zeng 1379* (FHMU 930); same location, 17 August 2013, *N.K. Zeng 1414* (FHMU 959); same location, 16 August 2014, *N.K. Zeng 1633* (FHMU 2493). Hainan Province: Dongfang County, Exian Mountain, elev. 633 m, 5 October 2014, *N.K. Zeng 1808* (FHMU 2494).

###### Notes.

Our molecular phylogenetic analyses indicate that the new collections and the holotype of *Ch.radiatus*, a species first described from Hunan Province of central China, group together with a strong statistical support based on a two-locus dataset (28S + *tef1*) (data not shown). This indicates that our specimens should be recognized as *Ch.radiatus* (Zhang et al. 2015). This species is new to Fujian and Hainan Province. Zhang et al. (2015) reported *Ch.radiatus* from under *Cunninghamialanceolata* (Lamb.) Hook, *Cyclobalanopsis* spp. and *Castanopsis* spp. We found the species associated with *Pinus* spp.

#### *Lanmaoa* G. Wu & Zhu L. Yang

*Lanmaoa*, typified by *L.asiatica* G. Wu & Zhu L. Yang, was erected recently. However, *Lanmaoa* and its closely related genus *Cyanoboletus* share overlapping morphological features and the most important diagnostic feature of *Lanmaoa* defined by Wu et al. (2016a) is not constant (Chai et al. 2018). Here, we treat *Lanmaoa* as an independent genus until the true taxonomic relationship between *Lanmaoa* and *Cyanoboletus* can be studied.

##### 5. Lanmaoa macrocarpa

Taxon classificationPlantaeBoletalesBoletaceae

N.K. Zeng, H. Chai & S. Jiang sp. nov.

MB828523

Figures 5a–c , 11

###### Typification.

CHINA. Hainan Province: Qiongzhong County, Yinggeling National Nature Reserve, elev. 750 m, 28 May 2017, *N.K. Zeng 3021* (FHMU 1982, holotype). GenBank accession numbers: 28S = MH879684, *tef1* = MH879713.

###### Etymology.

Latin, “*macrocarpa*”, meaning the new species has a large pileus.

###### Description.

*Basidiomata* large. *Pileus* 10–13 cm in diameter, subhemispherical when young, then convex to applanate; surface dry, finely tomentose, brownish red (8B6–9B6); context about 2.5 cm thick in the center of the pileus, yellowish, changing blue quickly when injured. *Hymenophore* poroid, depressed around apex of stipe; pores subround to angular, 1–2 mm in diameter, yellow (3A5), changing blue quickly, then turning brown slowly when injured; tubes about 1.5 cm in length. *Stipe* 8–11 × 1.5–2 cm, central, subcylindric, solid; surface dry, brownish red (9C6), sometimes reticulate at apex; context yellow, changing blue quickly when injured; basal mycelium yellowish (2A4). *Odor* indistinct.

*Basidia* 18–28 × 6–10 μm, clavate, thin-walled, colorless to yellowish in KOH; four-spored, sterigmata 3–4 μm in length. *Basidiospores* [40/2/2] (9–)10–12(–13) × 4.5–5 μm, Q=(2.00–)2.10–2.60(–2.67), *Q_m_*=2.39 ± 0.16, subfusoid and inequilateral in side view with a weak or distinct suprahilar depression, elliptic-fusiform to subfusiform in ventral view, slightly thick-walled (to 0.5 μm), olive-brown to yellowish brown in KOH, smooth. *Hymenophoral trama* boletoid; composed of colorless to yellowish in KOH, 4.5–9 μm wide, thin- to slightly thick-walled (to 0.5 μm) hyphae. *Cheilocystidia* 25–42 × 7–10 μm, ventricose, fusiform or subfusiform, thin-walled, yellowish in KOH, no encrustations. *Pleurocystidia* 25–45 × 7–11 μm, fusiform or subfusiform, thin-walled, yellowish in KOH, no encrustations. *Pileipellis* a trichoderm 120–160 μm thick, composed of rather vertically arranged, nearly colorless in KOH, 4.5–6 μm wide, thin-walled hyphae; terminal cells 21–32 × 4–6 μm long, clavate or subclavate, with obtuse apex. *Pileal trama* made up of hyphae 3–10 μm in diameter, thin-walled, nearly colorless in KOH. *Stipitipellis* hymeniform about 100 μm thick, composed of thin- to slightly thick-walled (to 0.5 μm) emergent hyphae, colorless in KOH, with clavate, subclavate, fusiform, or subfusiform terminal cells (22–43 × 3–9 μm), and occasionally with scattered clavate, 4-spored basidia. *Stipe trama* composed of longitudinally arranged, parallel hyphae 3–8 μm wide, cylindrical, thin- to slightly thick-walled (to 0.5 μm), yellowish in KOH. *Clamp connections* absent in all tissues.

**Figure 5.**
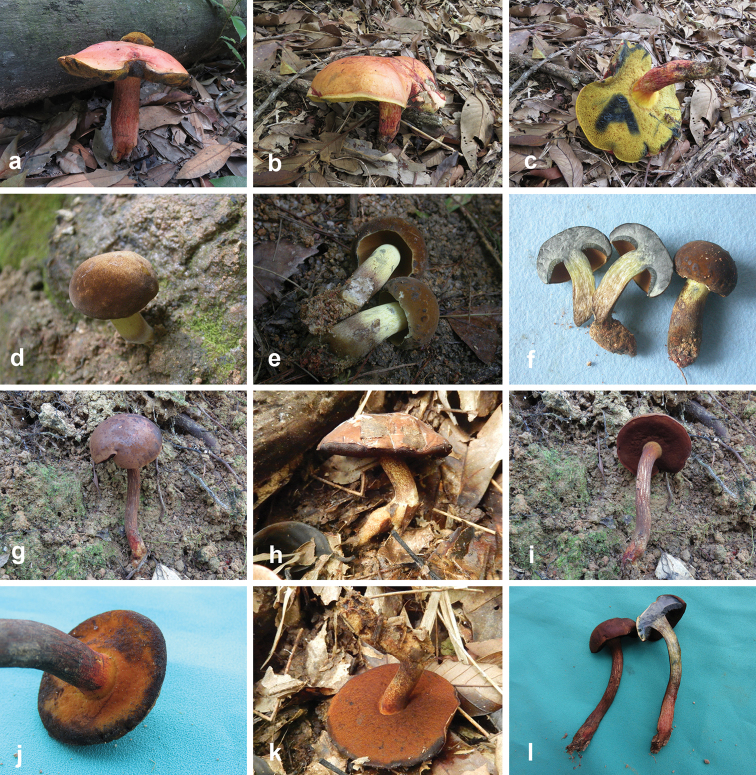
Basidiomata of boletes. **a–c***Lanmaoamacrocarpa* (a from FHMU 2212; **b–c** from FHMU 1982, holotype) **d–f***Neoboletushainanensis* (HKAS 90209) **g–l***Neoboletusmultipunctatus* (**g, i–j, l** from FHMU 2808 **h, k** from FHMU 1620, holotype). Photos by N.K. Zeng.

###### Habitat.

Solitary on the ground in forests dominated by *Castanopsiskawakamii* Hay. or *C.fissa* (Champ. ex Benth.) Rehd. et Wils.

###### Distribution.

Southeastern and southern China.

###### Additional specimens examined.

CHINA. Fujian Province: Sanming City, Geshikao National Forest Park, elev. 400 m, 16 August 2017, *N.K. Zeng* 3251 (FHMU 2212).

###### Note.

*Lanmaoamacrocarpa* is characterized by its large basidioma, brownish red pileus and stipe, thickness of hymenophore 3/5 times that of pileal context, and its association with *Castanopsis* spp. It is both morphologically similar and phylogenetically related to Chinese *L.rubriceps* N.K. Zeng & Hui Chai (Chai et al. 2018) and one collection tentatively named “*Lanmaoa* sp. HKAS 52518” (Fig. 3). However, *L.rubriceps* has a red to crimson, orange-red pileus, pores stuffed when young, sometimes tinged with reddish when old, and smaller basidiospores measuring 8–11 × 4–5 μm (Chai et al. 2018); careful examinations showed that *Lanmaoa* sp. HKAS 52518 has a smaller basidioma, a reddish to red or blackish-red pileus, and surface of stipe turning blue when injured.

#### *Neoboletus* Gelardi, Simonini & Vizzini

**Figure 6.**
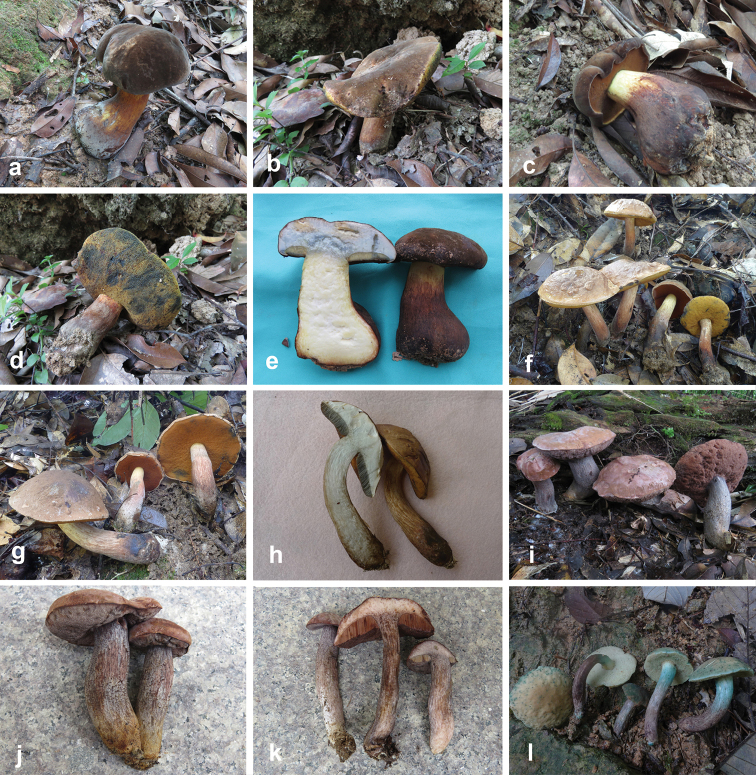
Basidiomata of boletes. **a–e***Neoboletusobscureumbrinus* (**a, e** from FHMU 2271 **b, d** from FHMU 2055 **c** from FHMU 2814) **f–h***Neoboletustomentulosus* (**h–i** from FHMU 842, **j** from FHMU 841) **i–k***Sutoriussubrufus* (FHMU 2004, holotype) **l***Tylopilusvirescens* (FHMU 1004). Photos by N.K. Zeng.

*Neoboletus*, typified by *N.luridiformis* (Rostk.) Gelardi et al., is characterized by stipitate-pileate or sequestrate; when basidiomata stipitate-pileate, pores brown, dark brown to reddish brown when young, becoming yellow when old (Fig. 6c, d, f), tubes always yellow (Figs 5f, l, 6e, h), hymenophore and context staining blue, and stipe usually covered with punctuations (Vizzini 2014; Wu et al. 2016a). The monophyly of *Neoboletus* has been assessed, and many species of the genus were described (Wu et al. 2014, 2016b). Astonishingly, the same authors recombined *Neoboletus* species in the genus *Sutorius* after a short time (Wu et al. 2016a). As a matter of fact, the stipe ornamentation pattern, spore print color, and colors of pores and tubes are fully different between the two genera (Halling et al. 2012; Vizzini 2014; Gelardi 2017). Furthermore, with more sequences added, our molecular data infers that *Neoboletus* forms an independent clade with strong support, and the genus *Sutorius* is sister to *Costatisporus*T.W. Henkel & M.E. Sm. (Smith et al. 2015) (Fig. 2). Thus, we recognize *Neoboletus* as an independent genus.

##### 6. Neoboletus hainanensis

Taxon classificationPlantaeBoletalesBoletaceae

(T.H. Li & M. Zang) N.K. Zeng, H. Chai & Zhi Q. Liang comb. nov.

MB828527

Figure 5d–f

Boletus hainanensis T.H. Li & M. Zang, Mycotaxon 80: 482, 2001
Sutorius hainanensis (T.H. Li & M. Zang) G. Wu & Zhu L. Yang, Fungal Diversity 81: 135, 2016

###### Habitat.

Solitary on the ground in forests dominated by fagaceous trees including *Lithocarpus* spp.

###### Distribution.

Southern and southwestern China.

###### Note.

*Boletushainanensis* T.H. Li & M. Zang was first described from Hainan Province of southern China (Zang et al. 2001). It was later also reported from Yunnan Province of southwestern China (Wu et al. 2016a) and was transferred to the genus *Sutorius*. It is called the “Black bolete” in Yunnan Province, and largely traded in local mushroom markets (Wang et al. 2004).

###### Specimens examined.

CHINA. Hainan Province: Changjiang County, Bawangling National Nature Reserve, elev. 650 m, 20 August 2009, *N.K. Zeng 523* (HKAS 90209). Yunnan Province: Kunming City, bought from market, 11 July 2015, *N.K. Zeng 2128* (FHMU 1392).

##### 7. Neoboletus multipunctatus

Taxon classificationPlantaeBoletalesBoletaceae

N.K. Zeng, H. Chai & S. Jiang sp. nov.

MB828528

Figures 5g–l , 12

###### Typification.

CHINA. Hainan Province: Qiongzhong County, Yinggeling National Nature Reserve, elev. 800 m, 3 August 2015, *N.K. Zeng* 2498 (FHMU 1620, holotype). GenBank accession numbers: 28S = MH879693, ITS = MH885354, *tef1* = MH879722.

###### Etymology.

Latin, “*multipunctatus*”, referring to the many punctuations on the stipe.

###### Description.

*Basidiomata* medium-sized. *Pileus* 5.7–7 cm in diameter, convex to applanate; surface dry, finely tomentose, brown (4D7), dark brown (5C7) to blackish brown (5D5); context 1–1.5 cm thick in the center of the pileus, yellowish (1A5), changing blue quickly when injured. *Hymenophore* poroid, depressed around apex of stipe; pores subround, 0.3–0.4 mm in diameter, brown (7B5) to reddish brown (6C8), changing bluish black quickly when injured; tubes 0.5–0.7 cm in length, yellowish (1A5), changing blue quickly when injured. *Stipe* 7–7.4 × 1–1.3 cm, central, subcylindric, solid, usually flexuous; surface dry, covered with reddish-brown (7B5) squamules; context yellow (1A3), changing blue (21B3) quickly when injured; basal mycelium yellow (1A3). *Odor* indistinct.

*Basidia* 27–37 × 6–10 μm, clavate, thin-walled, colorless to yellowish in KOH; four-spored, sterigmata 5–6 μm in length. *Basidiospores* [80/4/3] 8.5–11(–12) × 4–5 μm, Q=(1.80–)1.90–2.50(–2.75), *Q_m_*=2.22 ± 0.22, subfusoid and inequilateral in side view with a weak or distinct suprahilar depression, elliptic-fusiform to subfusiform in ventral view, slightly thick-walled (to 0.5 μm), olive-brown to yellowish brown in KOH, smooth. *Hymenophoral trama* boletoid; composed of colorless to yellowish in KOH, 4–8 μm wide, thin-walled hyphae. *Cheilocystidia* 27–34 × 5–7 μm, fusiform or subfusiform, thin-walled, fawn to tawny in KOH, no encrustations. *Pleurocystidia* 38–61 × 6–8 μm, fusiform or subfusiform, thin-walled, colorless to tawny in KOH, no encrustations. *Pileipellis* a trichoderm about 120 μm thick, composed of vertically arranged, nearly colorless to yellowish in KOH, 3–5 μm wide, thin-walled hyphae; terminal cells 21–70 × 3–5 μm, clavate or subclavate, with obtuse apex. *Pileal trama* made up of hyphae 3–8 μm in diameter, thin-walled, colorless to yellowish in KOH. *Stipitipellis* hymeniform about 100 μm thick, composed of thin-walled emergent hyphae, colorless to yellowish in KOH, with clavate, subclavate, fusiform or subfusiform terminal cells (25–44 × 3–9 μm), and occasionally with scattered clavate, 4-spored basidia. *Stipe trama* composed of longitudinally arranged, parallel hyphae 4–9 μm wide, cylindrical, thin to slightly thick-walled (to 0.5 μm), colorless in KOH. *Clamp connections* absent in all tissues.

###### Habitat.

Solitary on the ground in forests dominated by fagaceous trees including *Lithocarpus* spp.

###### Distribution.

Southern China.

###### Additional specimens examined.

CHINA. Hainan Province: Changjiang County, Bawangling National Nature Reserve, elev. 600 m, 22 August 2009, *N.K. Zeng 559* (HKAS 76851); Ledong County, Yinggeling National Nature Reserve, elev. 620 m, 6 May 2018, *N.K. Zeng 3324* (FHMU 2808).

###### Note.

*Neoboletusmultipunctatus* is characterized by a brown, dark brown to blackish brown pileus, brown to reddish-brown pores changing bluish black when injured, stipe surface densely covered with brown to reddish-brown punctuations, smaller basidiospores, and its association with fagaceous trees. It is both morphologically similar and phylogenetically related to *N.brunneissimus* (W.F. Chiu) Gelardi et al. (Fig. 2), a species originally described from Yunnan Province of southwestern China. However, *N.brunneissimus* has larger basidiospores measuring 10–14 × 4.5–5 μm, and it occurs in temperature regions in addition to subtropical belts (Wu et al. 2016a). *Neoboletusmultipunctatus* is also similar to *N.hainanensis* and *N.sinensis* (T.H. Li & M. Zang) Gelardi et al. morphologically. However, both pileal and stipe surface of *N.hainanensis* stain blue when injured, with white basal mycelium on the stipe, relatively larger basidiospores measuring 9.5–13.5 × 4–5 μm, and a trichodermium to ixotrichodermium pileipellis (Zang et al. 2001; Wu et al. 2016a). *Neoboletussinensis*, a species also described from Hainan Province, has a cherry red stipe with reticulations, larger basidiospores measuring 13–19 × 5–6.5 μm, and wider cystidia (Zang et al. 2001; Vizzini 2014).

##### 8. Neoboletus obscureumbrinus

Taxon classificationPlantaeBoletalesBoletaceae

(Hongo) N.K. Zeng, H. Chai & Zhi Q. Liang comb. nov.

MB828529

Figure 6a–e

Boletus obscureumbrinus Hongo, Mem. Fac. Lib. Arts. Educ. Shiga Univ. Nat. Sci., 18: 4, 1968
Sutorius obscureumbrinus (Hongo) G. Wu & Zhu L. Yang, Fungal Diversity 81: 138, 2016

###### Habitat.

Solitary or gregarious on the ground in forests dominated by fagaceous trees including *Lithocarpus* spp.

###### Distribution.

Southern and southwestern China; Japan (Hongo 1968).

###### Note.

*Boletusobscureumbrinus* Hongo was originally described from Japan (Hongo 1968) and later reported from Guangdong Province of southern China and Yunnan Province of southwestern China (Wu et al. 2016a). It was transferred to the genus *Sutorius* by Wu et al. (2016a); in the present study, we place the species in *Neoboletus* according to the evidence referred to above (Fig. 2). It is new to Hainan Province. The fruit body of this species is eaten by the Li people who live in the region (our own investigations).

###### Specimens examined.

CHINA. Hainan Province: Ledong County, Yinggeling National Nature Reserve, elev. 620 m, 5 June 2017, *N.K. Zeng 3091*, *3094, 3098* (FHMU 2052, 2055, 2059); same location, 6 May 2018, *N.K. Zeng 3310, 3353* (FHMU 2271, 2814).

##### 9. Neoboletus tomentulosus

Taxon classificationPlantaeBoletalesBoletaceae

(M. Zang, W.P. Liu & M.R. Hu) N.K. Zeng, H. Chai & Zhi Q. Liang comb. nov.

MB828530

Figure 6f–h

Boletus tomentulosus M. Zang, W.P. Liu & M.R. Hu, Acta Botanica Yunnanica 13: 150, 1991
Sutorius tomentulosus (M. Zang, W.P. Liu & M.R. Hu) G. Wu & Zhu L. Yang, Fungal Diversity 81: 142, 2016

###### Habitat.

Solitary or gregarious on the ground in forests dominated by *Castanopsiskawakamii* Hay.

###### Distribution.

Southeastern China.

###### Note.

*Boletustomentulosus* M. Zang et al. was first described from Fujian Province of southeastern China (Zang et al. 1991) and later reported from Guangdong Province of southern China (Wu et al. 2016a). Although the description of the protologue was brief (Zang et al. 1991), it has been well studied by Wu et al. (2016a). Our new collections were encountered near the type locality and augments our understanding of the species and the genus *Neoboletus*.

###### Specimens examined.

CHINA. Fujian Province: Zhangping County, Xinqiao Town, Chengkou Village, elev. 350 m, 27 July 2013, *N.K. Zeng 1285, 1286* (FHMU 841, 842).

#### *Sutorius* Halling, Nuhn & N.A. Fechner

*Sutorius*, typified by *S.eximius* (Peck) Halling et al., is mainly characterized by pores and tissues that are tinged with reddish at all growth stages, tissues not stained blue, a reddish-brown spore print, and transversely scissurate scales on stipe surface (Smith and Thiers 1971; Halling et al. 2012). Until now, only two taxa, *S.australiensis* (Bougher & Thiers) Halling and N.A. Fechner, and *S.eximius* (Peck) Halling et al., were described, excluding those in Wu et al (2016a). Herein, we describe another species new to science.

##### 10. Sutorius subrufus

Taxon classificationPlantaeBoletalesBoletaceae

N.K. Zeng, H. Chai & S. Jiang sp. nov.

MB828531

Figures 6i–k , 13

###### Typification.

CHINA. Hainan Province: Qiongzhong County, Yinggeling National Nature Reserve, elev. 850 m, 29 May 2017, *N.K. Zeng 3043* (FHMU 2004, holotype).

GenBank accession numbers: 28S = MH879698, ITS = MH885360, *tef1* = MH879728, *rpb2* = MH879745.

###### Etymology.

Latin, “*subrufus*” refers to the stipe surface and context of the species turning reddish when injured.

###### Description.

*Basidiomata* medium to large. *Pileus* 5–10 cm in diameter, subhemispherical to convex when young, then applanate; surface dry, finely tomentose, brown to pale reddish brown (10C2–11C3); context about 1.6 cm thick in the center of the pileus, white (6A1), changing reddish (9C3) when injured. *Hymenophore* poroid, adnate or slightly depressed around apex of stipe; pores angular, about 0.3 mm in diameter, pale brown (8C3), brown (7E2) to pale reddish brown (10C2), mostly unchanging in color when injured, but sometimes changing reddish; tubes about 1 cm in length, pale brown (8D3), unchanging in color when injured, but sometimes changing reddish. *Stipe* 6–10 × 1–2.2 cm, central, subcylindric, solid; surface dry, gray-white, but brownish yellow at base, covered with pale reddish-brown (7B2) to blackish-brown squamules, usually changing reddish when injured; context white (1D1–2), changing reddish (9C3) when injured; annulus absent; basal mycelium white (1A1). *Odor* indistinct.

*Basidia* 18–30 × 6–9 μm, clavate, thin-walled, colorless to yellowish in KOH; four-spored, sterigmata 2–3 μm in length. *Basidiospores* [200/24/3] (8–)9–12(–13.5) × 3.5–4.5 μm, Q=(2.25–)2.50–3.00(–3.29), *Q_m_*=2.79 ± 0.21, subfusoid and inequilateral in side view with a weak or distinct suprahilar depression, elliptic-fusiform to subfusiform in ventral view, slightly thick-walled (to 0.5 μm), olive-brown to yellowish brown in KOH, smooth. *Hymenophoral trama* boletoid; composed of colorless to yellowish in KOH, 5–10 μm wide, thin- to slightly thick-walled (up to 0.5 μm) hyphae. *Cheilocystidia* 28–45 × 7–10 μm, ventricose, fusiform or subfusiform, thin-walled, colorless to yellowish in KOH, no encrustations. *Pleurocystidia* 35–50 × 7–10 μm, fusiform or subfusiform, thin-walled, colorless to yellowish in KOH, no encrustations. *Pileipellis* a trichoderm about 100–150 μm thick, composed of rather vertically arranged, yellowish in KOH, 3.5–6 μm wide, thin-walled hyphae; terminal cells 30–43 × 3.5–6 μm, clavate or subclavate, with obtuse apex. *Pileal trama* made up of hyphae 4.5–10 μm in diameter, thin-walled, nearly colorless in KOH. *Stipitipellis* hymeniform about 60–80 μm thick, composed of thin-walled emergent hyphae, colorless in KOH, with clavate, subclavate terminal cells (22–28 × 4–9 μm), and occasionally with scattered clavate, four-spored basidia. *Stipe trama* composed of longitudinally arranged, parallel hyphae 4–8 μm wide, cylindrical, thin- to slightly thick-walled (to 0.5 μm), fawn to tawny in KOH, parallel hyphae. *Clamp connections* absent in all tissues.

###### Habitat.

Scattered, gregarious or caespitose on the ground in forests dominated by fagaceous trees, including *Lithocarpus* spp.

###### Distribution.

Southern China.

###### Additional specimens examined.

CHINA. Hainan Province: Qiongzhong County, Yinggeling National Nature Reserve, elev. 860 m, 29 May 2017, *N.K. Zeng 3045* (FHMU 2006); Ledong County, Yinggeling National Nature Reserve, elev. 650 m, 27 July 2017, *N.K. Zeng* 3140 (FHMU 2101).

###### Note.

*Sutoriussubrufus* is characterized by a brown to pale reddish-brown pileus, stipe surface and context turning reddish when injured, relatively smaller basidiospores, and it is restricted in tropical China. It is both morphologically similar and phylogenetically related to *S.eximius* (Peck) Halling et al. and *S.australiensis* (Bougher & Thiers) Halling and N.A. Fechner. However, stipe surface and context of *S.eximius* does not change when injured. Moreover, *S.eximius* has larger basidiospores, and a distribution in North and Central America (Singer 1947; Smith and Thiers 1971; Halling et al. 2012); *S.australiensis* has relatively larger basidiospores, a distribution in Australia, and is associated with Myrtaceae and Casuarinaceae (Halling et al. 2012).

#### *Tylopilus* P. Karst.

*Tylopilus*, typified by *T.felleus* (Bull.) P. Karst., is characterized by the pallid, pinkish, vinaceous and pinkish-brown hymenophore, white to pallid context without color change, but some species becoming rufescent or sea-green when injured, and the bitter taste of the context (Baroni and Both 1998; Henkel 1999; Fulgenzi et al. 2007; Osmundson and Halling 2010; Wu et al. 2016a; Magnago et al. 2017; Liang et al. 2018). In China, although lots of species of the genus have been previously discovered (Li et al. 2002; Fu et al. 2006; Gelardi et al. 2015; Wu et al. 2016a; Liang et al. 2018), still there are a large number of undescribed taxa in this region.

##### 11. Tylopilus virescens

Taxon classificationPlantaeBoletalesBoletaceae

(Har. Takah. & Taneyama) N.K. Zeng, H. Chai & Zhi Q. Liang comb. nov.

MB828532

Figure 6l

Boletus virescens Har. Takah. & Taneyama, The fungal flora in southwestern Japan, agarics and boletes 1: 45, 2016
Tylopilus callainus N.K. Zeng, Zhi Q. Liang & M.S. Su, Phytotaxa 343 (3): 271, 2018

###### Habitat.

Solitary or gregarious on the ground in forests dominated by fagaceous trees including *Lithocarpus* spp. or *Castanopsiskawakamii* Hay.

###### Distribution.

Southeastern and southern China; Japan (Terashima et al. 2016).

###### Note.

*Tylopiluscallainus* N.K. Zeng et al. was described from the south of China (Liang et al. 2018). This taxon was previously thought to be different from *B.virescens* Har. Takah. & Taneyama, a species described from Japan (Terashima et al. 2016). After a careful re-evaluation of specimens, we now know that the two taxa are conspecific, and *T.callainus* is synonymized with *B.virescens*. Clarifying the taxonomic relationship between the two taxa also indicated that the *B.virescens* is a member of *Tylopilus*, and thus the new combination is proposed. Illustrations and a full description have been provided by Liang et al. (2018).

###### Specimens examined.

CHINA. Fujian Province: Zhangping County, Xinqiao Town, Chengkou Village, elev. 350 m, 22 August 2013, *N.K. Zeng 1360, 1459* (FHMU2812, 1001); same location, 23 August 2013, *N.K. Zeng 1460* (FHMU 2813); same location, 24 August 2013, *N.K. Zeng 1464* (FHMU 1004). Hainan Province: Baisha County, Yinggeling National Nature Reserve, elev. 550 m, 1 August 2015, *N.K. Zeng 2436* (FHMU 1562); same location, 26 May 2017, *N.K. Zeng 2982* (FHMU 1943); same location, 27 May 2017, *N.K. Zeng 3001* (FHMU 1962); Ledong County, Jianfengling National Nature Reserve, elev. 850 m, 27 June 2018, *N.K. Zeng 3426, 3431* (FHMU 2810, 2811).

#### New combinations

According to the analytical results presented here, the following new combinations are proposed:

***Neoboletusferrugineus* (G. Wu, F. Li & Zhu L. Yang) N.K. Zeng, H. Chai & Zhi Q. Liang, comb. nov.**

MycoBank: MB828533

*Sutoriusferrugineus* G. Wu, Fang Li & Zhu L. Yang, Fungal Diversity 81: 134, 2016

***Neoboletusflavidus* (G. Wu & Zhu L. Yang) N.K. Zeng, H. Chai & Zhi Q. Liang, comb. nov.**

MycoBank: MB828534

*Sutoriusflavidus* G. Wu & Zhu L. Yang, Fungal Diversity 81: 135, 2016

***Neoboletusrubriporus* (G. Wu & Zhu L. Yang) N.K. Zeng, H. Chai & Zhi Q. Liang, comb. nov.**

MycoBank: MB828535

*Sutoriusrubriporus* G. Wu & Zhu L. Yang, Fungal Diversity 81: 139, 2016

***Neoboletussanguineoides* (G. Wu & Zhu L. Yang) N.K. Zeng, H. Chai & Zhi Q. Liang, comb. nov.**

MycoBank: MB828536

*Sutoriussanguineoides* G. Wu & Zhu L. Yang, Fungal Diversity 81: 140, 2016

***Neoboletussanguineus* (G. Wu & Zhu L. Yang) N.K. Zeng, H. Chai & Zhi Q. Liang, comb. nov.**

MycoBank: MB828537

*Sutoriussanguineus* G. Wu & Zhu L. Yang, Fungal Diversity 81: 141, 2016

## Discussion

Molecular phylogenetic analyses have been used widely to define the genera of boletes, and as a result, many genera were erected or merged (Zeng et al. 2012, 2014b; Nuhn et al. 2013; Wu et al. 2014, 2016a, b). Recently, the genus *Neoboletus* was synonymized with *Sutorius* solely based on the evidence of molecular data (Wu et al. 2016a). Our molecular phylogenetic analyses based on a four-locus dataset (28S + ITS + *tef1* + *rpb*2) with sequences from taxa of *Neoboletus*, *Sutorius*, *Costatisporus*, and *Caloboletus* (Fig. 2) indicate those species that morphologically match the concept of genus *Neoboletus* do not belong in *Sutorius*; instead, they form an independent clade with strong support (Fig. 2). At the same time, the morphological features including the stipe ornamentation pattern, spore print color, and color change of tissues are different between the two genera and has been noted in previous studies (Halling et al. 2012; Gelardi 2017). It is noteworthy that the color of tubes of *Neoboletus* is always yellow (Figs 5f, l, 6e, h), and in this genus the pores usually become yellow when old (Fig. 6d, f), whereas the color of tubes and pores of *Sutorius* are always tinged with reddish at different growth stages (Fig. 6i–k).

The present study further shows that the most important diagnostic feature of the genus *Lanmaoa*, viz. “short hymenophoral tubes (thickness of hymenophore 1/3–1/5 times that of pileal context at the position halfway to the pileus center) and a slow color change when injured” defined by Wu et al. (2016b) is not constant (Chai et al. 2018), for the thickness of hymenophore is about 3/5 times that of pileal context in our newly described *L.macrocarpa*. Additionally, context and hymenophore of our new species turn quickly and strongly when injured (Fig. 5c).

According to current molecular data, 10 lineages (lineages 1–10) of *Sutorius* were found (Fig. 2). Lineages 4 and 6 were identified as *S.australiensis* and *S.eximius* respectively in a previous study (Halling et al. 2012). Lineages 1, 2, 3, 5, 7 and 9 may have not diverged enough (Fig. 2) and are treated here as a series of closely related taxa or disjunct populations of previously described entities; these will be assessed in the future with more DNA sequences and more collections. As to lineages 8 and 10, they should be treated as independent taxa due to their high degree divergence. Moreover, morphological and ecological features (described above) of specimens (FHMU 2004, FHMU 2006, FHMU 2101) in lineage 8 from Hainan Province are also different from the described taxa of *Sutorius*, and thus, the new taxon *S.subrufus* was proposed. Lineage 10 was not described due to the paucity of the materials (Halling et al. 2012).

Subtropical and tropical China is believed to be a biodiversity hotspot. Mycologists have paid much attention to boletes of the region in the past decade, and many taxa have been discovered (Bi et al. 1997; Zeng and Yang 2011; Zeng et al. 2012, 2013, 2014a, b, 2015a, b, 2016, 2017, 2018; Zang 2013; Liang et al.2016, 2017, 2018; Chai et al. 2018; Xue et al. 2018). Among of them, many have been found to be as North American or European species (Bi et al. 1997; Zang 2013), and recent studies have shown that species shared between subtropical/tropical China and North America/Europe are rare but that there are many common species between Japan and subtropical/tropical China (Zeng et al. 2013, 2016, 2017). Our study now reveals that the geographic distributions of the Japanese *C.guanyui*, *N.obscureumbrinus*, and *T.virescens* extend into subtropical or tropical China.

**Figure 7.**
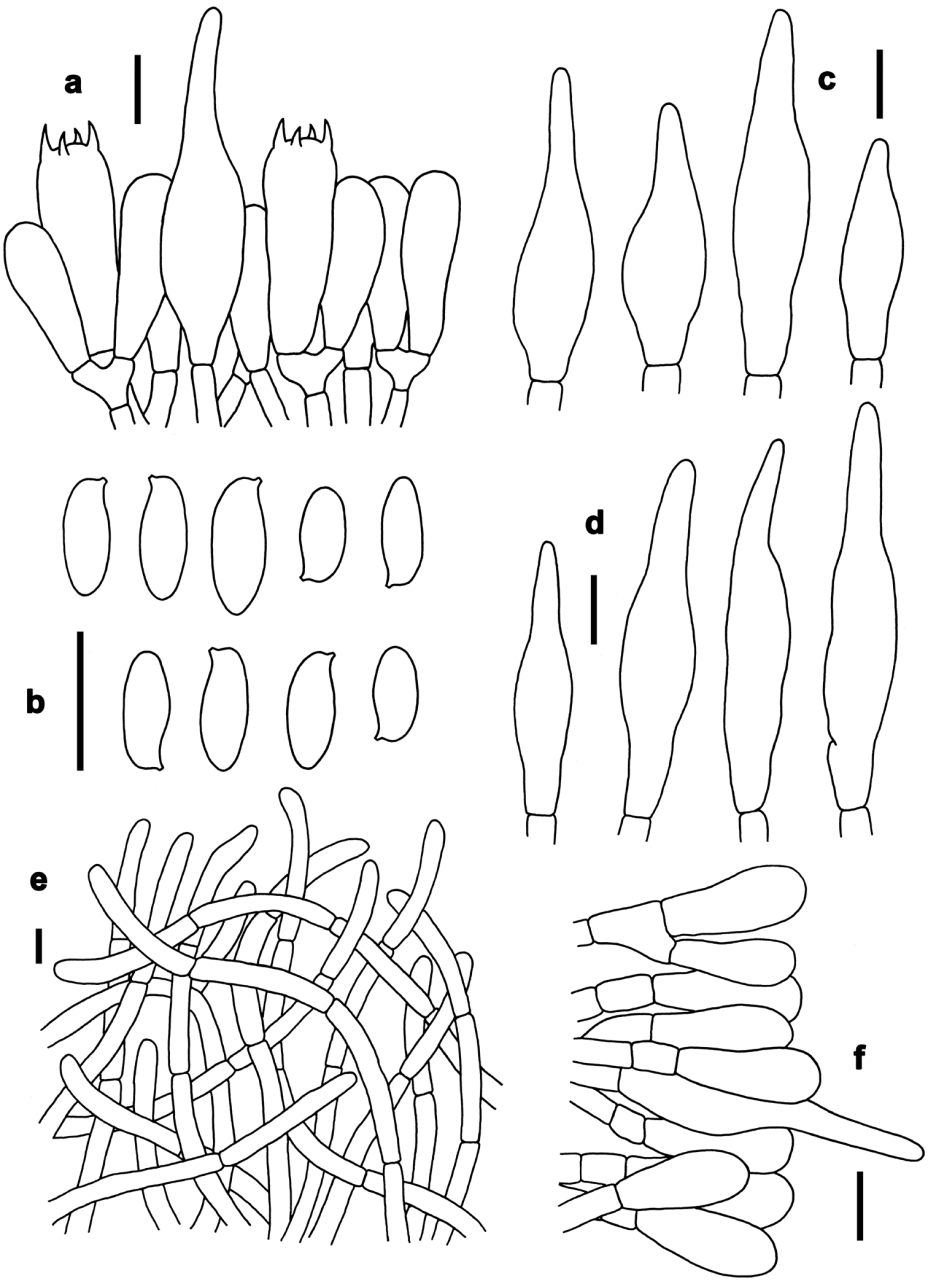
Microscopic features of *Butyriboletushuangnianlaii* (FHMU 2207, holotype). **a** Basidia and pleurocystidium **b** Basidiospores **c** Cheilocystidia **d** Pleurocystidia **e** Pileipellis **f** Stipitipellis. Scale bars: 10 μm.

**Figure 8.**
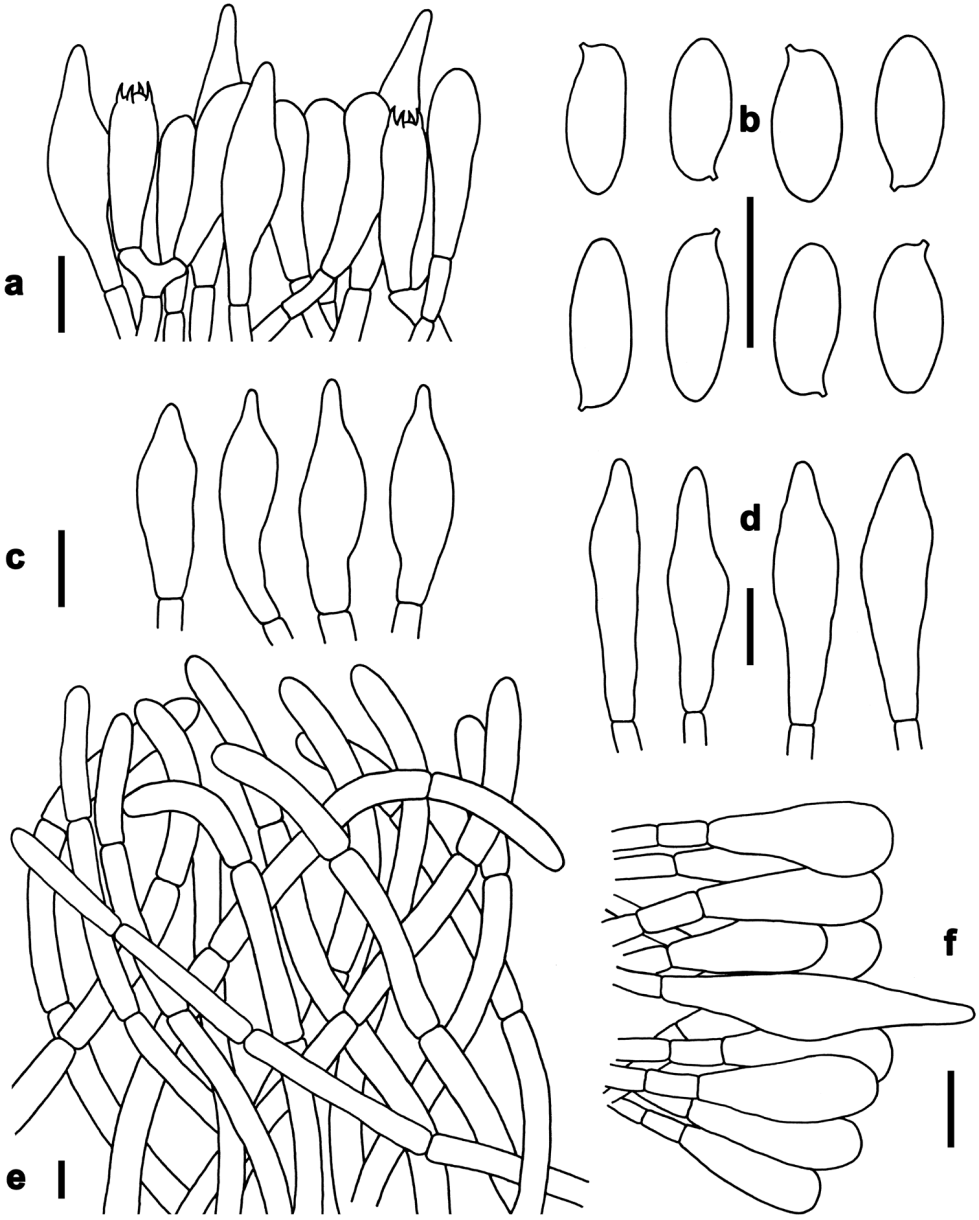
Microscopic features of *Caloboletusguanyui* (FHMU 2040). **a** Basidia and pleurocystidia **b** Basidiospores **c** Cheilocystidia **d** Pleurocystidia **e** Pileipellis **f** Stipitipellis. Scale bars: 10 μm.

**Figure 9.**
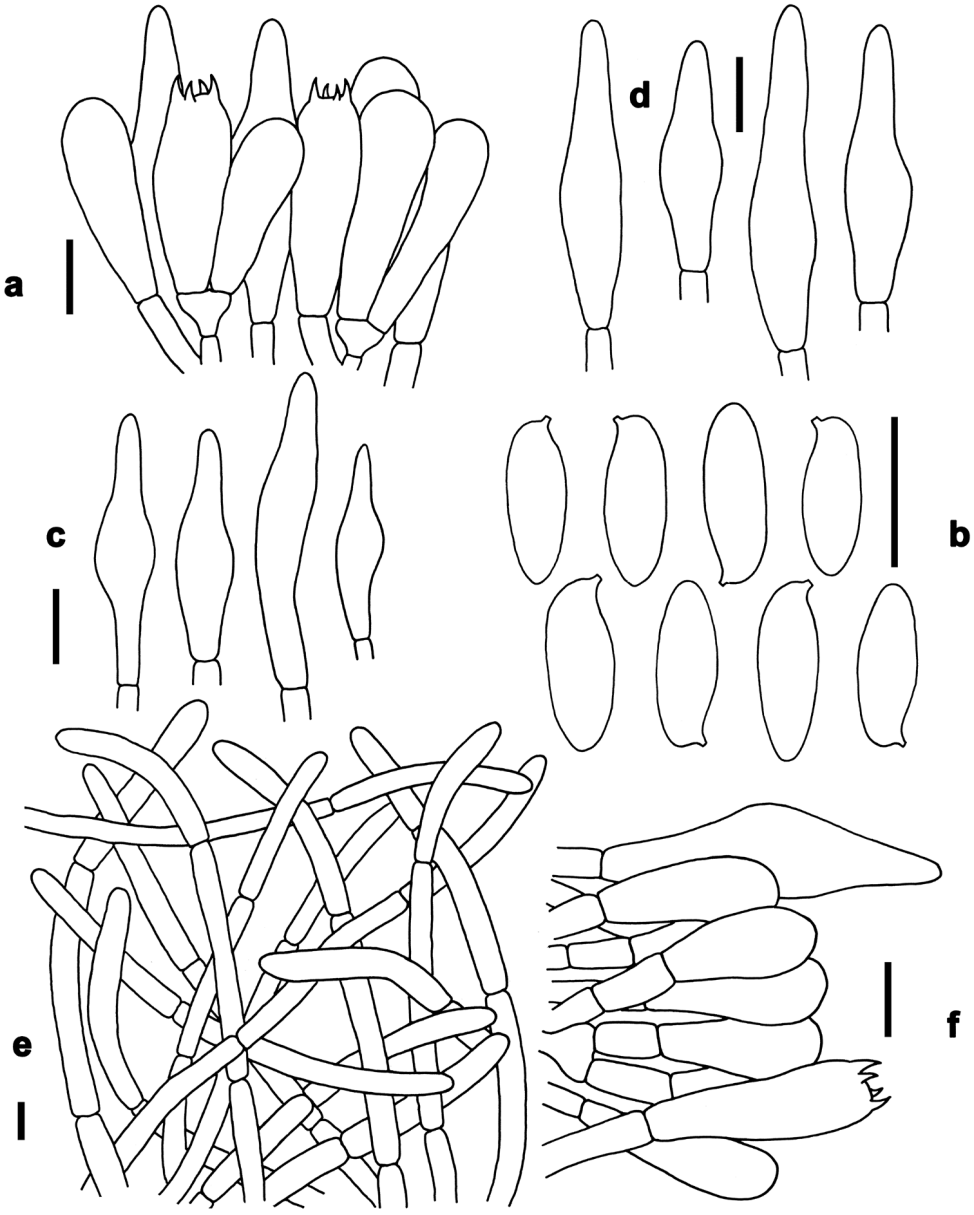
Microscopic features of *Caloboletusxiangtoushanensis* (FHMU 883). **a** Basidia and pleurocystidia **b** Basidiospores **c** Cheilocystidia **d** Pleurocystidia **e** Pileipellis **f** Stipitipellis. Scale bars: 10 μm.

**Figure 10.**
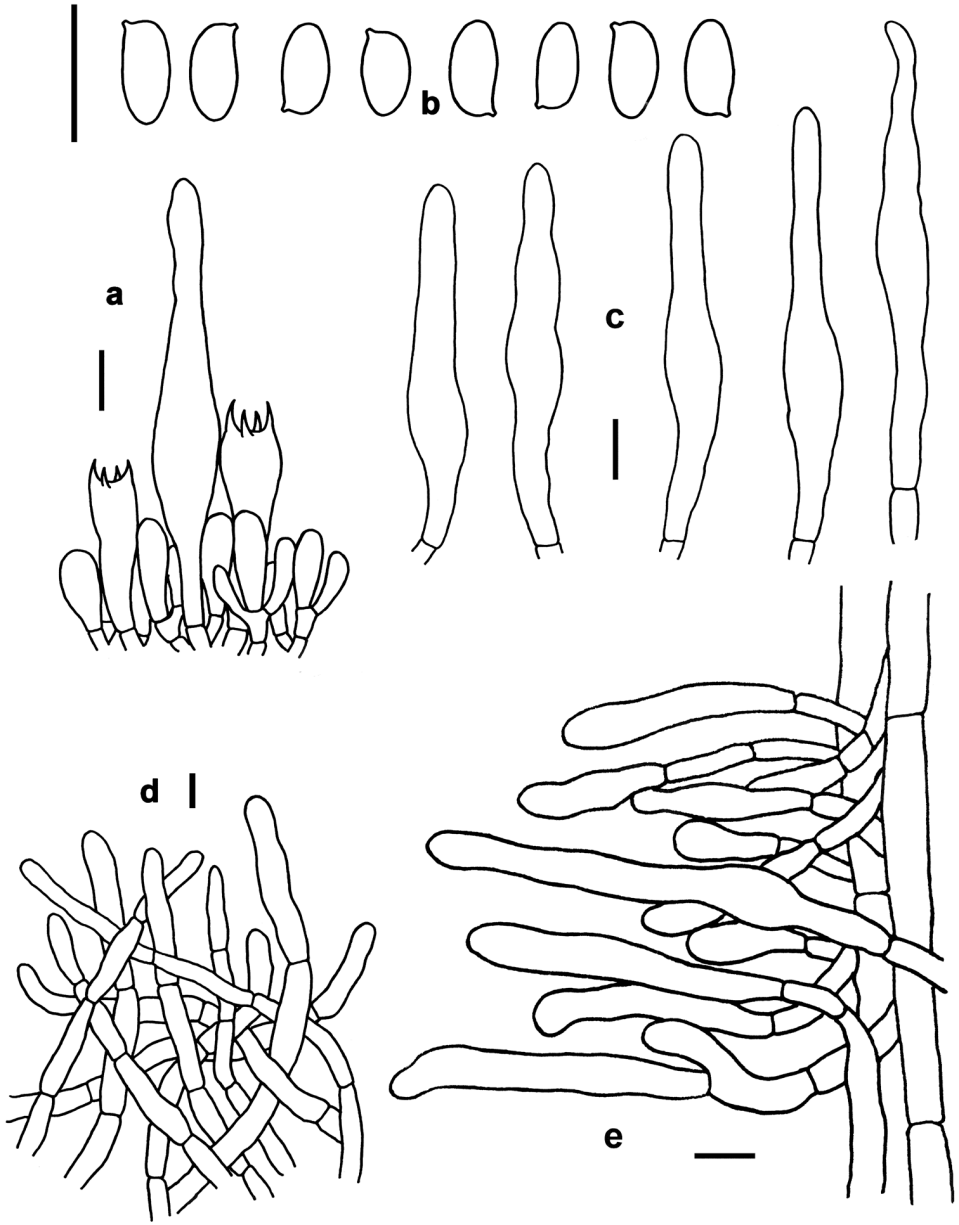
Microscopic features of *Chalciporusradiatus* (FHMU 930). **a** Basidia and pleurocystidium **b** Basidiospores **c** Cheilocystidia **d** Pileipellis **e** Stipitipellis. Scale bars: 10 μm.

**Figure 11.**
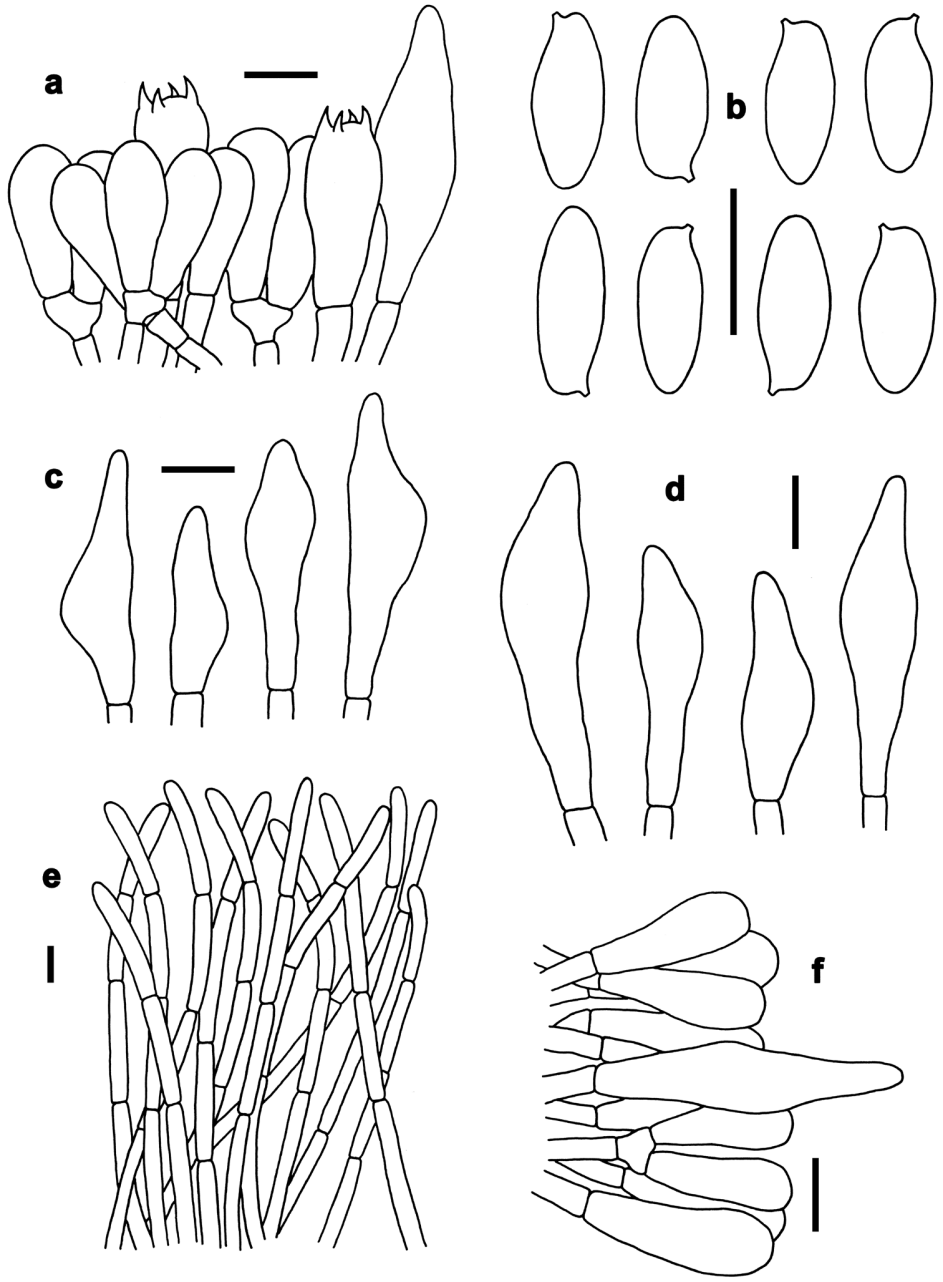
Microscopic features of *Lanmaoamacrocarpa* (**a–e** from FHMU 1982, holotype **f** from FHMU 2212). **a** Basidia and pleurocystidium **b** Basidiospores **c** Cheilocystidia **d** Pleurocystidia **e** Pileipellis **f** Stipitipellis. Scale bars: 10 μm.

**Figure 12.**
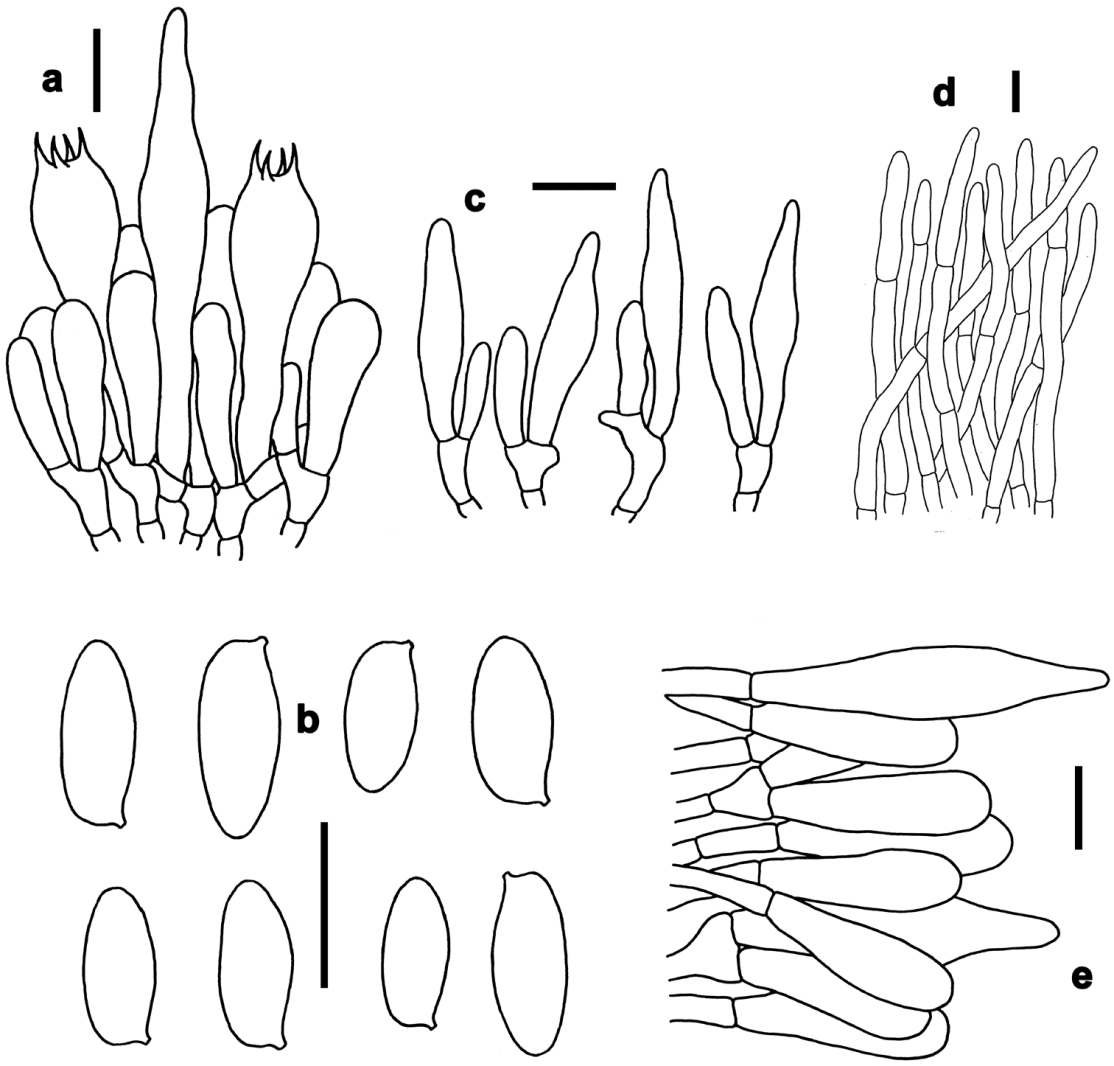
Microscopic features of *Neoboletusmultipunctatus* (FHMU 1620, holotype). **a** Basidia and pleurocystidium **b** Basidiospores **c** Cheilocystidia **d** Pileipellis **e** Stipitipellis. Scale bars: 10 μm.

**Figure 13.**
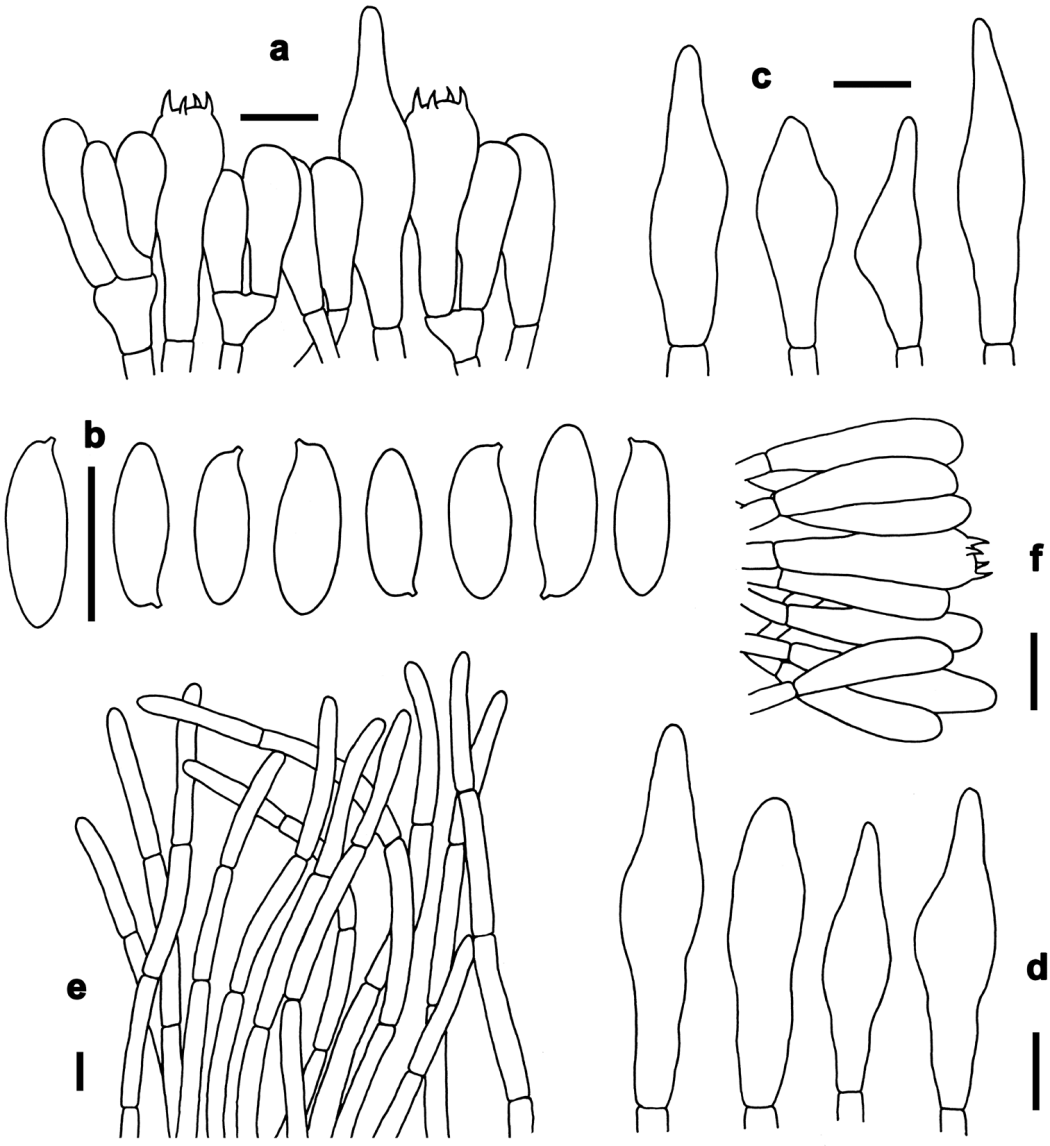
Microscopic features of *Sutoriussubrufus* (FHMU 2004, holotype). **a** Basidia and pleurocystidium **b** Basidiospores **c** Cheilocystidia **d** Pleurocystidia **e** Pileipellis **f** Stipitipellis. Scale bars: 10 μm.

## Supplementary Material

XML Treatment for Butyriboletus huangnianlaii

XML Treatment for Caloboletus guanyui

XML Treatment for Caloboletus xiangtoushanensis

XML Treatment for Chalciporus radiatus

XML Treatment for Lanmaoa macrocarpa

XML Treatment for Neoboletus hainanensis

XML Treatment for Neoboletus multipunctatus

XML Treatment for Neoboletus obscureumbrinus

XML Treatment for Neoboletus tomentulosus

XML Treatment for Sutorius subrufus

XML Treatment for Tylopilus virescens
